# Development of super-specific epigenome editing by targeted allele-specific DNA methylation

**DOI:** 10.1186/s13072-023-00515-5

**Published:** 2023-10-21

**Authors:** Nivethika Rajaram, Alexandra G. Kouroukli, Susanne Bens, Pavel Bashtrykov, Albert Jeltsch

**Affiliations:** 1 Institute of Biochemistry and Technical Biochemistry, Department of Biochemistry, University of Stuttgart, Allmandring 31, 70569 Stuttgart, Germany; 2 Institute of Human Genetics, University of Ulm and Ulm University Medical Center, Albert-Einstein-Allee 11, 89081 Ulm, Germany

**Keywords:** Epigenome editing, Targeted DNA methylation, dCas9, DNMT3A/3L, Allele discrimination, Specificity

## Abstract

**Background:**

Epigenome editing refers to the targeted reprogramming of genomic loci using an EpiEditor which may consist of an sgRNA/dCas9 complex that recruits DNMT3A/3L to the target locus. Methylation of the locus can lead to a modulation of gene expression. Allele-specific DNA methylation (ASM) refers to the targeted methylation delivery only to one allele of a locus. In the context of diseases caused by a dominant mutation, the selective DNA methylation of the mutant allele could be used to repress its expression but retain the functionality of the normal gene.

**Results:**

To set up allele-specific targeted DNA methylation, target regions were selected from hypomethylated CGIs bearing a heterozygous SNP in their promoters in the HEK293 cell line. We aimed at delivering maximum DNA methylation with highest allelic specificity in the targeted regions. Placing SNPs in the PAM or seed regions of the sgRNA, we designed 24 different sgRNAs targeting single alleles in 14 different gene loci. We achieved efficient ASM in multiple cases, such as ISG15, MSH6, GPD1L, MRPL52, PDE8A, NARF, DAP3, and GSPT1, which in best cases led to five to tenfold stronger average DNA methylation at the on-target allele and absolute differences in the DNA methylation gain at on- and off-target alleles of > 50%. In general, loci with the allele discriminatory SNP positioned in the PAM region showed higher success rate of ASM and better specificity. Highest DNA methylation was observed on day 3 after transfection followed by a gradual decline. In selected cases, ASM was stable up to 11 days in HEK293 cells and it led up to a 3.6-fold change in allelic expression ratios.

**Conclusions:**

We successfully delivered ASM at multiple genomic loci with high specificity, efficiency and stability. This form of super-specific epigenome editing could find applications in the treatment of diseases caused by dominant mutations, because it allows silencing of the mutant allele without repression of the expression of the normal allele thereby minimizing potential side-effects of the treatment.

**Supplementary Information:**

The online version contains supplementary material available at 10.1186/s13072-023-00515-5.

## Background

The genetic material of human cells is stored in the cell nucleus in the form of chromatin comprising the DNA wrapped around histone proteins and a complex ensemble of accessory proteins. Chromatin plays structural and regulatory functions, which are defined by complex patterns of post-translational modifications of histone tails, methylation of DNA and non-coding RNAs. These chromatin signals together are often termed the epigenome [1]. Dozens of chromatin-modifying and interacting enzymes and protein complexes, writers, erasers and readers, are involved in a setting of a cell-type specific epigenome, which controls gene activity and is essential for embryonic development, cellular differentiation, and adaptation of cells to environmental conditions [2–4].

The dynamic nature of the epigenome inspired the development of methods for targeted epigenome editing [5–9]. The main aim of this technique is to set a desired chromatin state at a target genomic locus in an artificial manner to control gene expression or other chromatin functions. Writing repressive chromatin marks can lead to the inactivation of an actively transcribed target gene, and, vice versa, deposition of activating chromatin marks can trigger transcription of a silenced gene. Epigenome editing is currently used for fundamental research and is a promising direction for the development of targeted therapy in personalized medicine. The key tool of epigenome editing is an EpiEditor, a designed protein assembled from two functionally distinct units: a targeting and an editing unit. The targeting unit binds to the DNA sequence specifically allowing to deliver the EpiEditor to the targeted genomic locus. The editing unit possesses a catalytic activity required to deposit the desired chromatin mark or it recruits endogenous enzymes able to deposit the mark. Various chromatin modifying enzymes have been characterized and applied as editing units of EpiEditors. For example, initial work has used DNMT3A [10] and SUV39H1 [11] fused to zinc finger proteins to silence genes via DNA methylation and methylation of lysine 9 on histone 3, respectively. Later, a catalytically deactivated version of the Cas9 protein (dCas9) was introduced as targeting unit [12]. It can recognize DNA sequence directly by forming Watson–Crick base pairs with a short 20-mer RNA, the single-guide RNA (sgRNA), generating an RNA–DNA hybrid at the target site. An additional requirement for the interaction of the sgRNA/dCas9 complex with the DNA is the presence of trinucleotide proto-spacer adjacent motif (PAM) directly at the 3' end of the target DNA sequence, which is NGG in the case of dCas9 (where N stands for any nucleotide). In the current work the dCas9 targeting unit was combined with a DNMT3A catalytic domain fused to a DNMT3L C-terminal domain as editing unit [13]. The DNMT3A subunit contained an R887E exchange that was shown to increase the specificity of targeted DNA methylation by suppression of untargeted activity [14]. Both entities were connected by a SunTag allowing to recruit up to 10 DNMT3A/3L units to each sgRNA/dCas9 complex [15]. This leads to signal amplification and supports the dimerization of the DNMT3A/3L unit that is required for the formation of a catalytically active enzyme complex [16].

One of the key performance parameters of EpiEditors is the editing specificity and, in this context, both functional units contribute to the final outcome. The targeting unit has to be designed to bind only the desired genomic locus and control the activity of the editing unit to deliver the modification only at this particular site and nowhere else in the genome. The most commonly used epigenome editing scenario is the silencing or activation of a selected gene by setting corresponding chromatin marks at its gene regulatory elements. Since most genes are present in two copies in the human genome, such an approach would result in simultaneous modulation of the expression of both alleles. However, there are cases where interrogation of only a single allele, i.e. an allele-specific epigenome editing (ASEE), might be beneficial. One potential clinical application could be an epigenetic inactivation of a dominant mutant allele in disorders such as Huntington's disease [17], Machado–Joseph disease [18], or Frontotemporal dementia [19], without affecting the second wild type allele. ASEE has two prerequisites, the presence of a sequence variation at the target site in one allele and the ability of the EpiEditor targeting unit to recognize one allele only. According to the International HapMap project, two human genomes differ from each other at every 1000 bases [20]. The most frequent variations are single-nucleotide polymorphisms (SNPs), which occur on a population level with a frequency of one per 300 bases. Statistically, some SNPs are in heterozygous states in individuals and allow for the discrimination of alleles. In some instances, disease causing mutations which could be used to address ASEE are found in the promoter regions of genes, e.g. in case of the C228T mutation in the TERT core promoter that stimulates its expression and contributes to cancer formation [21]. In other cases, heterozygous SNPs that are not pathogenic by themselves may be identified in gene promoters, and allow selective silencing of one allele, which contains additional disease-causing SNPs in the gene body.

In this work, we aimed to apply the sgRNA/dCas9 unit for the development of a universal ASEE system able to introduce allele-specific DNA methylation (ASM). Therefore, we systematically explored if and to what extent SNPs in the seed region of the sgRNA/dCas9 complex or in the PAM motif can be used to deliver DNA methylation specifically to one allele of the target locus. ASM was evaluated with respect to locus and allele-specificity, stability and its effects on allele-specific gene silencing. Our data show that strong ASM could be achieved in many of the test cases, where loci with the discriminatory SNP positioned in the PAM region in general showed higher success rate and better specificity. In selected cases, ASM was stable up to 11 days in HEK293 cells and it led up to a 3.6-fold change in allelic expression ratios. These results suggest that ASM might have clinical applications in future aiming towards the selective silencing of dominant disease alleles without affecting the expression of the second copy of the gene.

## Results

### Target selection and transient transfection of HEK293

**Fig. 1:**
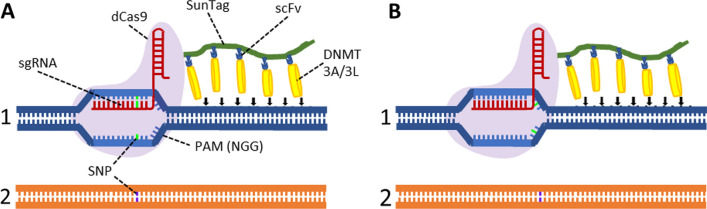
Scheme illustrating the selective binding of the EpiEditor complex to the target allele. **A** Sequence discrimination by a SNP in the sgRNA seed region. The sequence of allele 1 matches the sgRNA leading to the DNA methylation delivery in the targeted allele. Allele 2 has a nucleotide mismatch in the seed region of the sgRNA, which disfavors the complex assembly. **B** Sequence discrimination by a SNP in the PAM site. The sequence of allele 1 matches the sgRNA and it has a PAM at the distal end. Hence, the EpiEditor complex assembles at allele 1 leading to the DNA methylation deposition. Although the sgRNA matches the sequence of allele 2, the SNP disrupts the PAM and the absence of the PAM prevents binding of the sgRNA/dCas9 complex

HEK293 cells were chosen as the model cell line for allele-specific DNA methylation experiments because of their easy handling and good transfection yields. Information on heterozygous SNPs in HEK293 cells were extracted from a public database [22]. An in-silico search was performed to identify the SNPs in the promoter region of genes. As next filtering step, DNA methylation levels were taken from the MBD2-pulldown sequencing data of a previous work [23], and we focused on SNPs in unmethylated promoter CGIs. Previous work has shown that the DNA binding of sgRNA/Cas9 complexes is most dependent on the correct base pairing in the 3′ region of the sgRNA (7–12 bps, called “seed” region) [24–26]. Therefore, in the next step, the search results were filtered regarding the presence of SNPs in a potential PAM site or next to a PAM site in a distance of up to 7 bps from the 3' end of a potential sgRNA. Finally, from many potential candidates, 14 target genes containing suitable SNPs were selected. In each case, up to 3 sgRNAs were designed to target only one allele as shown in Table 1. The detailed targeting strategy in each experiment is shown in Additional file 1. Based on the position of the SNP in the sgRNA or PAM region, the 24 individual experiments could be divided into three categories, either with the SNP in the sgRNA seed region (12 experiments), at position 2 of the NGG PAM sequence (5 experiments), or at PAM position 3 (7 experiments) (Fig. 1, Table 1). SNPs in the PAM region leading to G > Y changes were preferred (where Y stands for C or T), as these changes were demonstrated to allow best discrimination of dCas9 binding [27, 28].

**Table 1:** Compilation of ASM experiments conducted in this study

No.	Gene	SNP location	Experiment	SNP	Target allele	ASM (Δ%)	ASM ratio
1	DAP3	PAM3	DAP3-PAM3	T to G	G	44	4.4
2	DAP3	Seed	DAP3-Seed1	T to G	G	15	1.4
3	DAP3	Seed	DAP3-Seed2	T to G	T	27	5.6
4	GPD1L	PAM3	GPD1L-PAM3	G to T	G	50	7.6
5	GSPT1	PAM3	GSPT1-PAM3	T to G	G	47	7.1
6	GSPT1	Seed	GSPT1-Seed1	T to G	G	18	1.7
7	GSPT1	Seed	GSPT1-Seed2	T to G	T	28	2.1
8	ISG15	PAM2	ISG15-PAM2	C to G	G	63	8.5
9	ISG15	Seed	ISG15-Seed1	C to G	G	36	2.3
10	ISG15	Seed	ISG15-Seed2	C to G	C	34	1.8
11	MAPK1	Seed	MAPK1-Seed1	T to G	T	19	1.4
12	MRPL52	PAM3	MRPL52-PAM3	G to T	G	52	3.7
13	MSH6	PAM3	MSH6-PAM3	T to G	G	46	10
14	MYH10	Seed	MYH10-Seed1	G to A	A	16	2.6
15	NARF	PAM2	NARF-PAM2	G to T	G	45	5.6
16	NARF	Seed	NARF-Seed1	G to T	G	43	3.0
17	NARF	Seed	NARF-Seed 2	G to T	T	31	1.7
18	PDE8A	PAM2	PDE8A-PAM2	G to T	G	19	3.1
19	PDE8A	Seed	PDE8A-Seed1	G to T	G	26	1.9
20	PDE8A	Seed	PDE8A-Seed2	G to T	T	45	4.4
21	RAF1	PAM3	RAF1-PAM3	G to A	G	4	1.7
22	RALB	PAM2	RALB-PAM2	G to C	G	8	2.6
23	TTC41P	PAM3	TTC41P-PAM3	G to T	G	6	2.3
24	TYK2	PAM2	TYK2-PAM2	G to C	G	15	2.7

“ASM (Δ%)” and “ASM (ratio)” refer to the difference and ratio of the increases in DNA methylation at the on- and off-target alleles. The detailed targeting strategy in each case including genome coordinates is shown in Additional file 1

To achieve targeted ASM, transient transfection of the plasmids co-expressing dCas9-10× SunTag with BFP, scFv-DNMT3A/3L with sfGFP, and sgRNA with DsRed was performed in HEK293 cells. Control experiments were conducted with a scrambled sgRNA that does not have a binding site in the human genome [29]. Initial studies showed that cells positive for all three plasmids exhibited highest fluorescence of the corresponding reporter proteins on day 3 post-transfection. Hence, fluorescence-activated cell sorting (FACS) of triple-positive cells was conducted at this time point. Genomic DNA was isolated from the FACS-sorted cells at day 3 after transfection or at later time points and subjected to bisulfite treatment. Library preparation was performed using the bisulfite-converted samples, followed by NGS and data analysis to investigate DNA methylation at the on-target and off-target alleles in the untransfected cells, and in cells after epigenome editing with the specific sgRNA or with the scrambled sgRNA (Additional file 2: Fig. S1). Additionally, off-target allele methylation was studied at the VEGFA locus that due to its open chromatin state has been shown to be highly accessible for recombinantly expressed DNMTs [14]. Most DNA methylation experiments were conducted in three independent biological replicates.

### Targeting ASM with a SNP in the seed region of the sgRNA

**Fig. 2:**
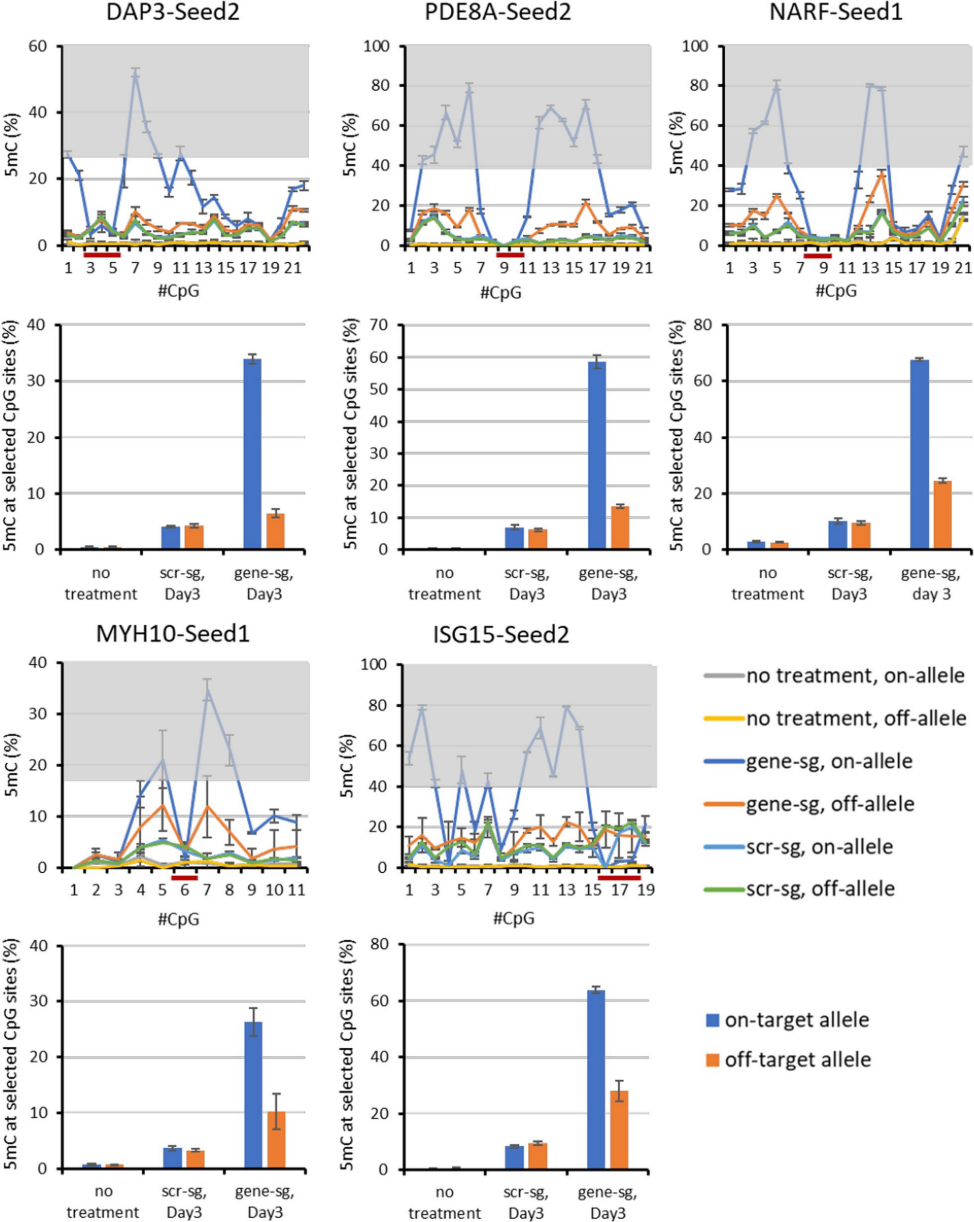
Examples of DNA methylation profiles and methylation levels observed after targeting a SNP in the seed region of the sgRNA. The graphs show the DNA methylation at each CpG site in the different target regions. The CpG sites in the grey regions were included for the calculation of the respective summary bar graphs. “scr-sg” refers to samples treated with scrambled sgRNA, “gene-sg” to samples treated with the allele-specific sgRNA. The sgRNA binding site is indicated by a dark red line. The bar graphs show corresponding levels of average DNA methylation at the selected CpG sites. Error bars show the standard deviation (SD) of three independent biological repeats except for ISG15-Seed2 and MYH10-Seed1 where two repeats were conducted

A total of 12 target genes were addressed by individual allele-specific sgRNAs with a SNP positioned in the seed region of the sgRNA. In each case, the targeted allele is referred to as the “on-target” allele, the second allele as “off-target”. In all cases, both alleles of the untransfected samples showed almost no DNA methylation, as expected after the pre-selection for unmethylated CGIs. Examples of experiments resulting in strongest ASM efficiency are shown in Fig. 2. In the cases of the DAP3-Seed2, PDE8A-Seed2 and NARF-Seed1 experiments, the transfected samples showed high DNA methylation of up to 80% at many CpG sites of the on-target allele. In each case, the DNA methylation data showed that binding of the dCas9 complex protected the sgRNA binding site from methylation (“sgRNA/dCas9 binding footprint”) as observed in previous studies [13, 14]. The off-target alleles in these experiments showed almost no to little DNA methylation increase. In all experiments with the scrambled sgRNA, very low DNA methylation levels were observed and there was no difference between the alleles indicating high target allele specificity in these cases. Methylation data observed in experiments with no or less efficient ASM are shown in Additional file 2: Fig. S2.

To summarize the data and compare the results of different experiments, DNA methylation levels of individual CpG sites were averaged. For this, CpG sites with DNA methylation levels of at least 50% of the highest methylation percentage delivered in the on-target allele were included (grey-shaded region in the DNA methylation profile graph of each target in Fig. 2). For uniformity in the analysis of the DNA methylation levels across different targets, this procedure was applied in each case. The same CpG sites were used to calculate the average DNA methylation levels of on-target and off-target allele DNA methylation in every sample. These analyses confirmed the previous conclusions showing high methylation at the on-target alleles, low methylation at the off-target alleles and low methylation of both allele after treatment with the scrambled sgRNA for DAP3-Seed2 and PDE8A-Seed2 (Fig. 2). In the case of NARF-Seed1, MYH10-Seed1 and ISG15-Seed2, the off-target allele methylation was little higher approaching about half of the methylation level of the on-target allele, indicating that the allele discrimination of the sgRNA/dCas9 complex was less efficient. In other cases, like DAP3-Seed1 (Additional file 2: Fig. S2) the allele specificity was even lower.

As an additional control of the specificity of the epigenome editing, methylation was also analyzed at the VEGFA locus containing 12 CpG sites as an off-target locus. The data revealed low off-target locus methylation levels of 5–10% in each case (Additional file 2: Fig. S3). These methylation levels correspond to the results at the target locus after treatment of the cells with scrambled sgRNAs. The methylation observed at off-target alleles after allele-specific editing and methylation at target alleles after treatment of cells with scrambled sgRNAs can be explained by an untargeted methylation activity of the DNMT3A/3L construct in these experiments, a problem that has been observed in previous work [14, 30, 31].

### Targeting ASM with a SNP positioned at the second position of the PAM

**Fig. 3:**
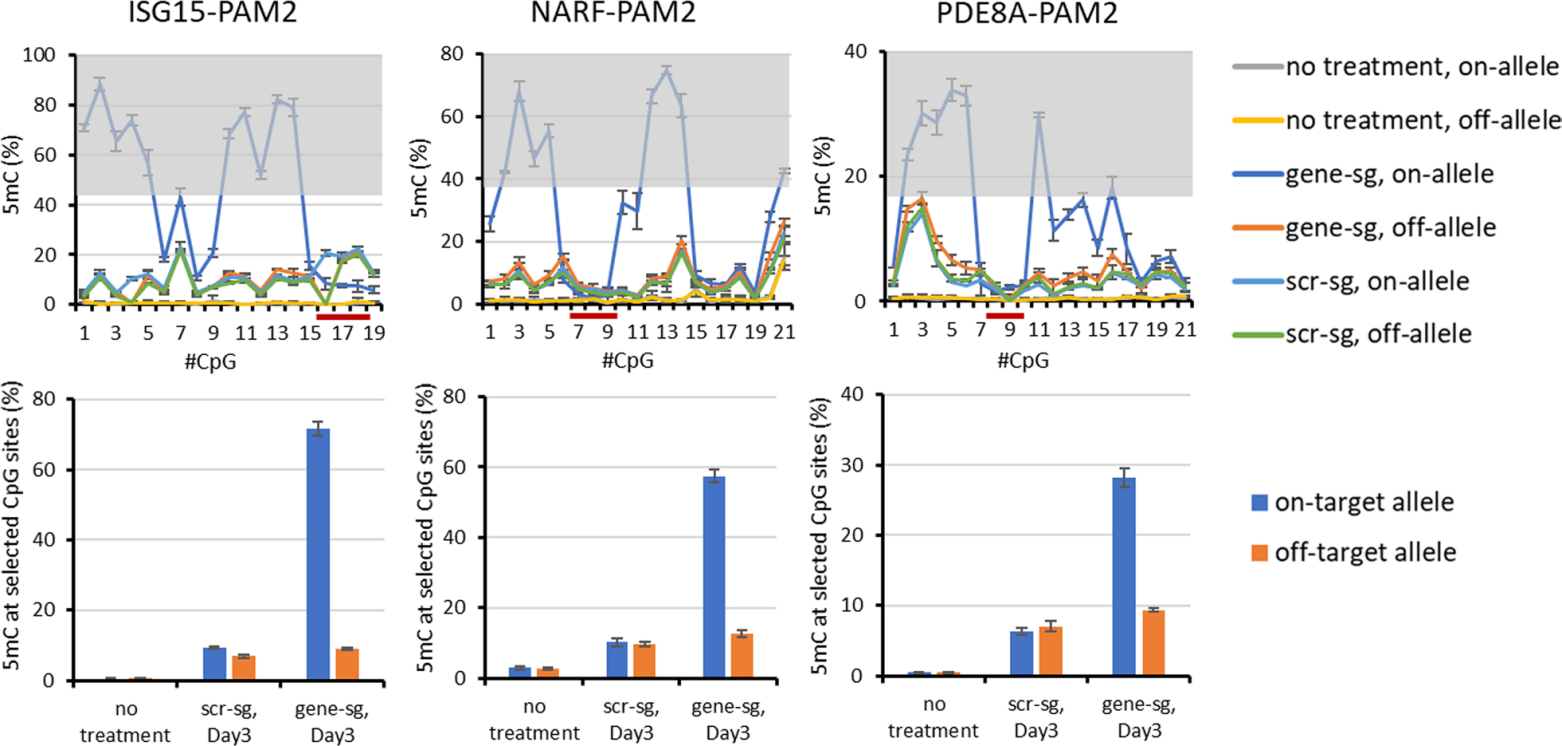
Examples of DNA methylation profiles and methylation levels observed after targeting a SNP in the second position of the PAM site. The graphs show the DNA methylation at each CpG site in the different target regions. The CpG sites in the grey regions were included for the calculation of the respective summary bar graphs. “scr-sg” refers to samples treated with scrambled sgRNA, “gene-sg” to samples treated with the allele-specific sgRNA. The sgRNA binding site is indicated by a dark red line. The bar graphs show corresponding levels of average DNA methylation at the selected CpG sites. Error bars show the standard deviation (SD) of three independent biological repeats

In the cases of PDE8A, NARF, ISG15, TYK2, and RALB gene loci, allele-specific targeting could be designed using an sgRNA that places the SNP in the second position of the PAM site. The on-target alleles of these genes contain the NGG PAM. The off-target alleles of PDE8A-PAM2 and NARF-PAM2 contain an NTG sequence, the off-target alleles of RALB-PAM2, TYK2-PAM2, and ISG15-PAM2 contain an NCG sequence. ASM was most successful in ISG15-PAM2, NARF-PAM2 and PDE8A-PAM2 (Fig. 3). In the cases of ISG15-PAM2 and NARF-PAM2, multiple CpG sites on the on-target allele showed over 70% DNA methylation deposition documenting very efficient allele targeting. With PDE8A-PAM2, on-target allele methylation levels were lower, but in all three cases the sgRNA/dCas9 complex showed a clear discrimination between the on-target and the off-target alleles indicating efficient introduction of ASM. Moreover, the sgRNA/dCas9 complex footprint was observed in the DNA methylation profile of the on-target alleles also indicating the specific DNA binding of the sgRNA/dCas9 complex. Off-target allele DNA methylation levels were comparable to the scrambled sgRNA controls and methylation levels observed at the VEGFA off-target locus control (Additional file 2: Fig. S3). The DNA methylation profiles of additional experiments using a SNP at the second PAM position for allele discrimination which led to weaker ASM are shown in Additional file 2: Fig. S4.

### Targeting ASM with a SNP positioned at the third position of the PAM

**Fig. 4:**
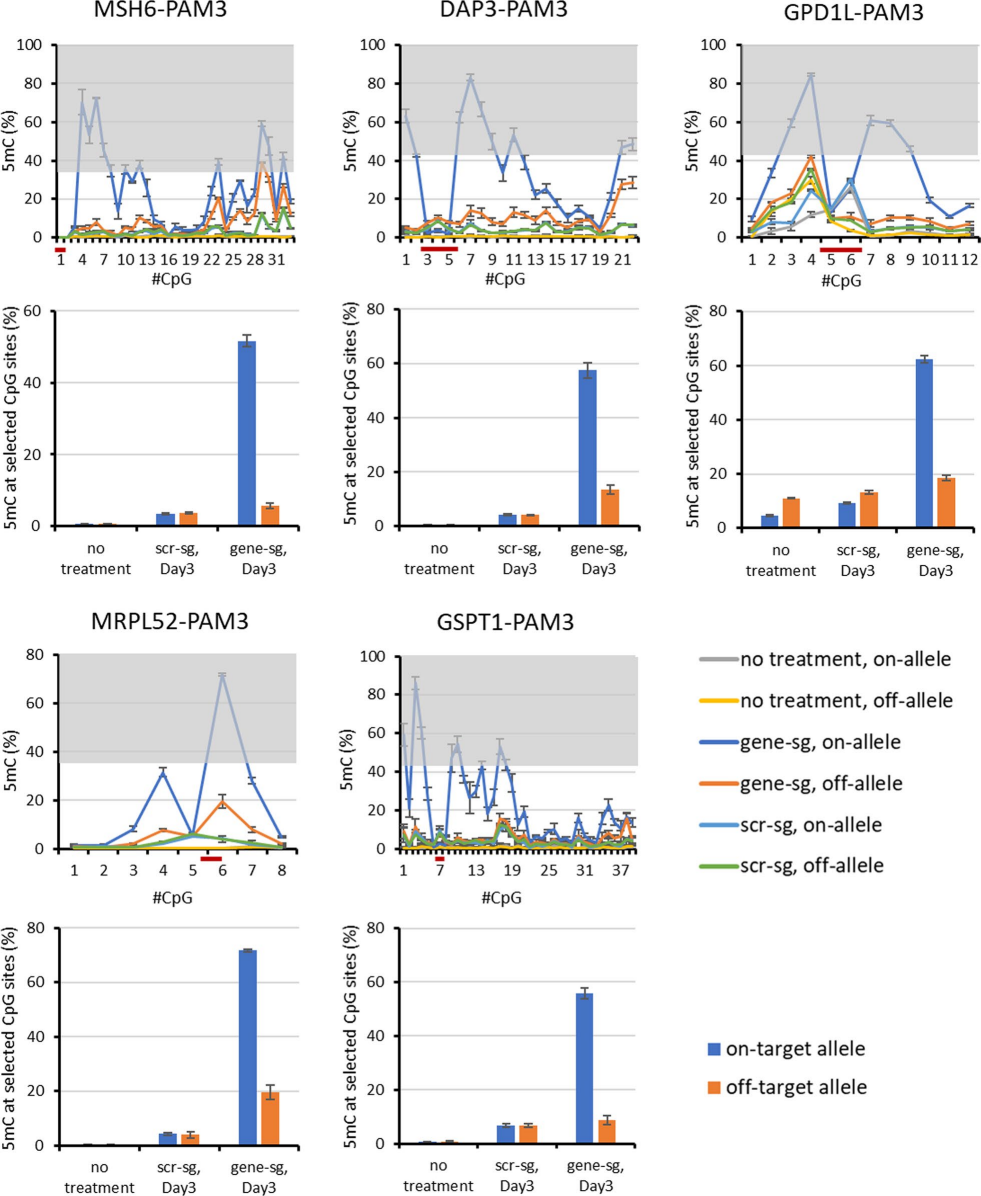
Examples of DNA methylation profiles and methylation levels observed after targeting a SNP in the third position of the PAM site. The graphs show the DNA methylation at each CpG site in the different target regions. The CpG sites in the grey regions were included for the calculation of the respective summary bar graphs. “scr-sg” refers to samples treated with scrambled sgRNA, “gene-sg” to samples treated with the allele-specific sgRNA. The sgRNA binding site is indicated by a dark red line. The bar graphs show corresponding levels of average DNA methylation at the selected CpG sites. Error bars show the SD of three independent biological repeats except for MSH6-PAM3 where two repeats were conducted

In the case of the MSH6, DAP3, GPD1L, MRPL52, GSPT1, RAF1 and TTC41P gene loci, sgRNAs could be designed that place the SNP at the third position of the PAM site. ASM was successfully implemented in case of MSH6-PAM3, DAP3-PAM3, GPD1L-PAM3, MRPL52-PAM3, and GSPT1-PAM3 (Fig. 4). In each case, high on-target allele DNA methylation and very good ASM specificity was observed as documented by low methylation levels of the samples treated with scrambled sgRNA (Fig. 4) and at the VEGFA off-target locus (Additional file 2: Fig. S3). The methylation of the off-target allele was as low as the controls in the cases of MSH6-PAM3 and GSPT1-PAM3 and slightly higher in case of DAP3-PAM3 and MRPL52-PAM3. In GDP1L-PAM3, the off-target allele showed slightly higher methylation in all samples including untreated cells. The DNA methylation profiles of additional experiments using a SNP at the third PAM position for allele discrimination which led to weaker ASM are shown in Additional file 2: Fig. S5.

### Comparison of ASM achieved at one genomic locus by different targeting methods

**Fig. 5:**
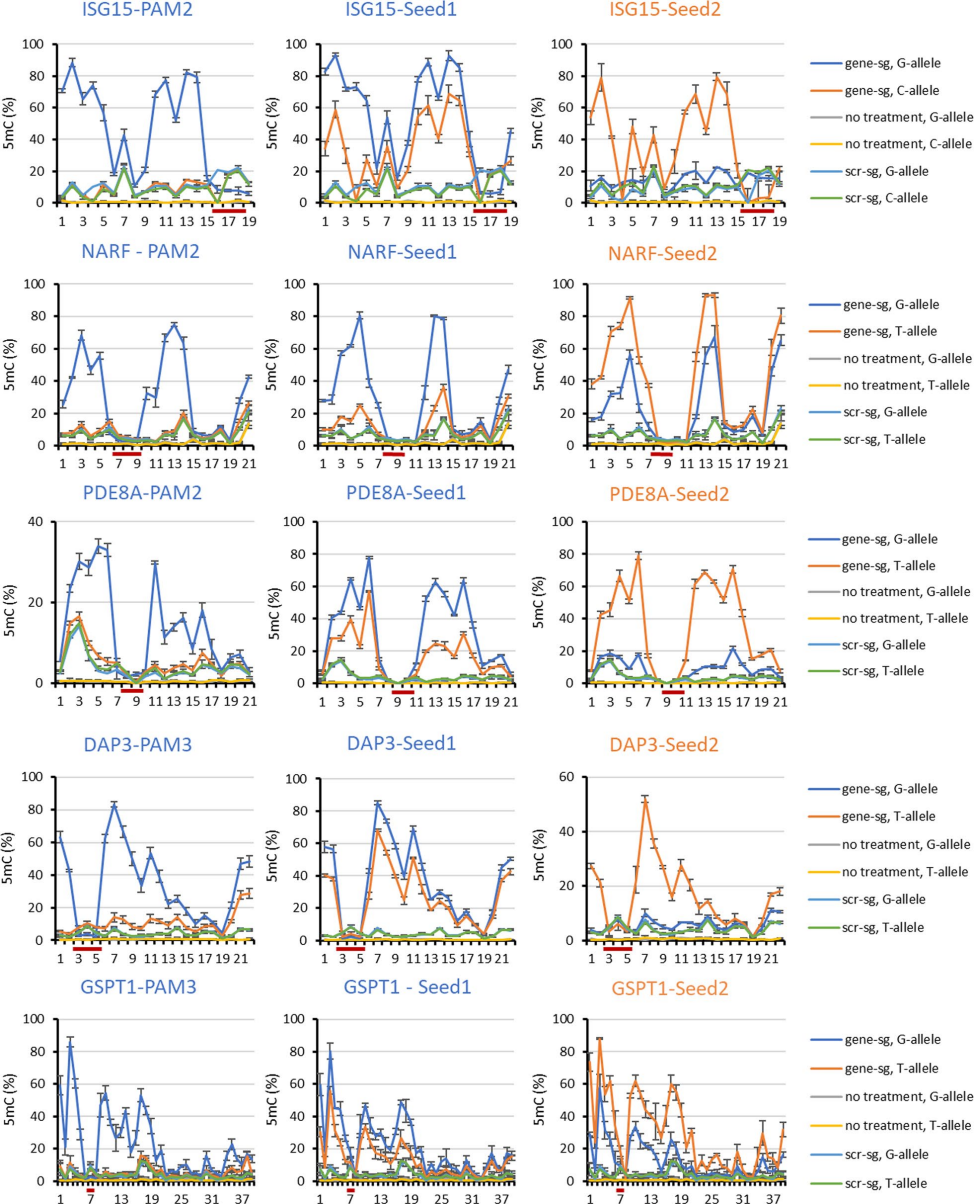
Compilation of ASM targeted by three different sgRNA, one with the SNP in the PAM region and two with the SNP in the seed region. The graphs show the DNA methylation at each CpG site in the different target regions. The x-axis indicates the CpG site number. “scr-sg” refers to samples treated with scrambled sgRNA, “gene-sg” refers to samples treated with the allele-specific sgRNA. The color of the heading indicates which allele was targeted. Error bars show the SD of three independent biological repeats except for ISG15-Seed2 which was conducted twice

For the 5 loci, ISG15, NARF, PDE8A, DAP3 and GSPT1, multiple sgRNA could be designed that place the SNP for allele discrimination either in the PAM or sgRNA seed region. Of note, if the SNP was present in the seed region, two distinct sgRNAs could be designed to address both alleles of the target gene. This was not possible if the SNP is in the PAM region, where the non-G allele cannot be targeted. Hence in all these cases, 3 different targeting constructs were used. The results of these experiments are presented in Fig. 5. Strikingly, in two examples, PDE8A and DAP3, efficient and opposite ASM was observed on both alleles of the target locus. In general, these data illustrate that ASM was more specific when the SNP was present in the PAM region.

### Stability of ASM in HEK293 cells

The stability of the introduced targeted DNA methylation is a critical issue and previous work has provided examples of high and low stability [23, 32–35]. Systematic studies showed that this depends (among other factors) on the genomic locus [23, 36]. To investigate the stability of the introduced ASM in our system, the DNA methylation of both alleles in the target regions in the ISG15-Seed2, MYH10-Seed1, PDE8A-PAM2, ISG15-PAM2, MRPL52-PAM3, MSH6-PAM3, and GPD1L-PAM3 experiments was studied at regular intervals after transfection. The day of transfection is noted as day 0 followed by the sorting of triple-positive cells on day 3. The sorted cells were seeded and the culture was maintained until day 11. A fraction of cells was collected on day 3, day 5, day 8, and day 11 and the DNA methylation was analyzed. To estimate the expression of the EpiEditors, we exploited the fact that fluorophores are co-expressed with the EpiEditors from the corresponding plasmids, namely BPF together with dCas9-10× SunTag, sfGFP with scFv-DNMT3A/3L, and DsRed with the sgRNAs. The fluorescence of the different cell population was analyzed, showing that the signals of the fluorophores co-expressed with the EpiEditors were strongly reduced at day 8 and vanished at day 11 (Additional file 2: Fig. S6). As these fluorescent proteins are known to be very stable, we conclude that around day 8 the cellular contents of sgRNA, scFv-DNMT3A/3L and dCas9-10× SunTag will be very low and active deposition of DNA methylation must have stopped.

**Fig. 6:**
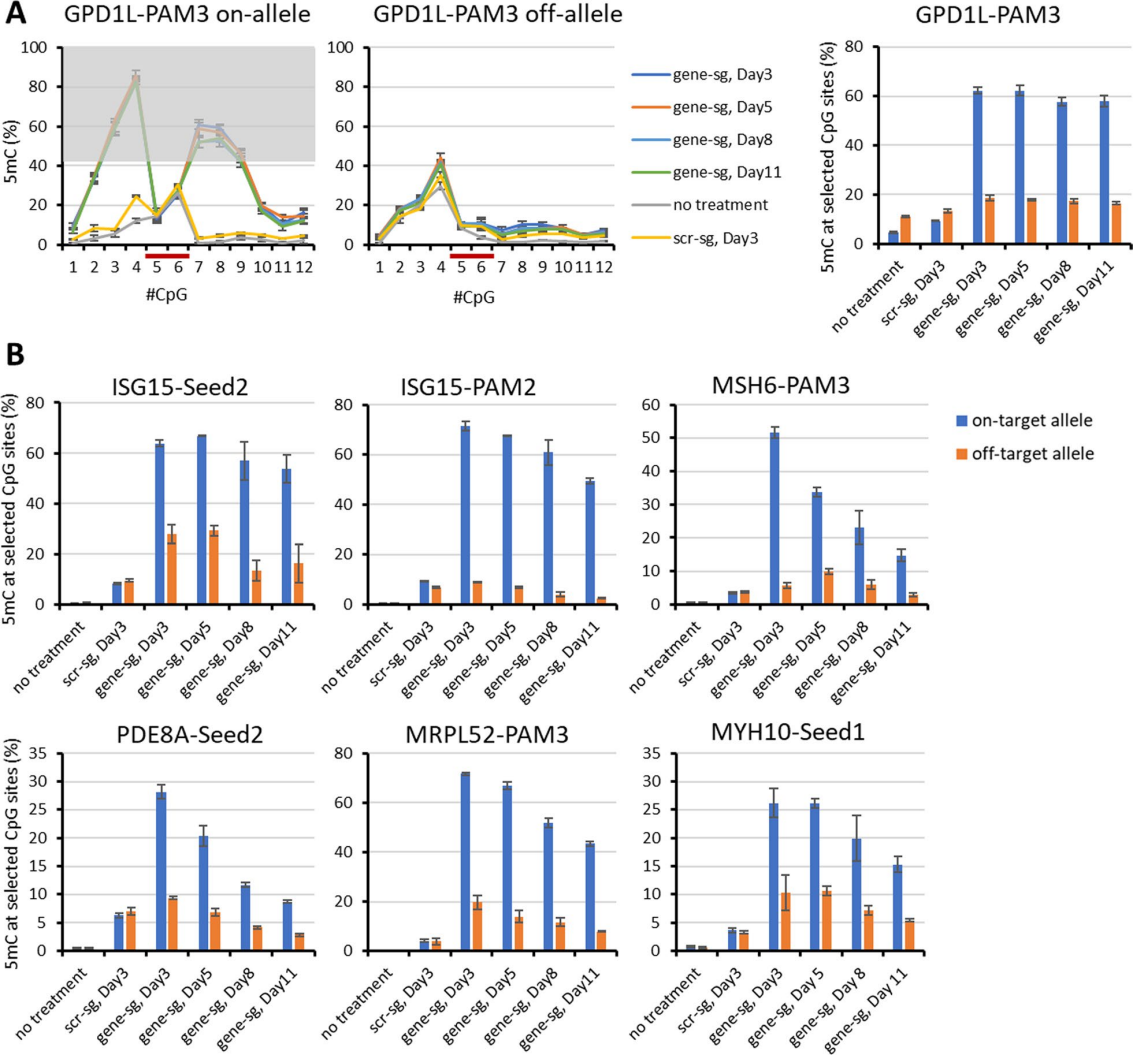
Stability of ASM. **A** Exemplary DNA methylation profiles of GPD1L-PAM3 on-target and off-target alleles on the days 3, 5, 8 and 11 after transfection. The bar graph indicates the average DNA methylation levels at the selected CpG sites. Error bars show the SD of three biological repeats. “scr-sg” refers to samples treated with scrambled sgRNA, “gene-sg” refers to samples treated with the allele-specific sgRNA. **B** Bar graphs indicating the average DNA methylation levels at the selected CpG sites of ISG15-Seed2, MSH6-PAM3, ISG15-PAM2, MHY10-Seed1, MRPL52-PAM3 and PDE8A-PAM2 on day 3, 5, 8 and 11 after transfection. Error bars show the SD of three independent biological repeats except for except for ISG15-Seed2, MSH6-PAM3 and MYH10 Seed1 which were conducted twice. Corresponding DNA methylation profiles are shown in Additional file 2: Fig. S6

The methylation data of the corresponding samples showed that every target region had a maximum of ASM on day 3 followed by a gradual decrease of DNA methylation until day 11 although the loss of DNA methylation showed different kinetics (Fig. 6, Additional file 2: Fig. S7). The experiments GPD1L-PAM3 and ISG15-Seed2 delivered ASM with high stability of the DNA methylation, and on day 11, DNA methylation levels corresponding to 93% and 84%, respectively, of the methylation observed on day 3 were still present. These results indicate that the methylation was propagated in cells at least for some days even in the absence of the EpiEditors. In the experiments ISG15-PAM2, MRPL52-PAM3, and MYH10-Seed1, 69%, 60% and 57%, respectively, of the DNA methylation deposited on day 3 was retained. Other experiments such as PDE8A-PAM3 and MSH6-PAM3 showed low stability of ASM and retained only about 30% of the DNA methylation observed on day 3 until day 11 indicating that the stability of ASM is locus dependent as expected [23].

### Modulation of allele-specific gene expression ratios by ASM

**Fig. 7:**
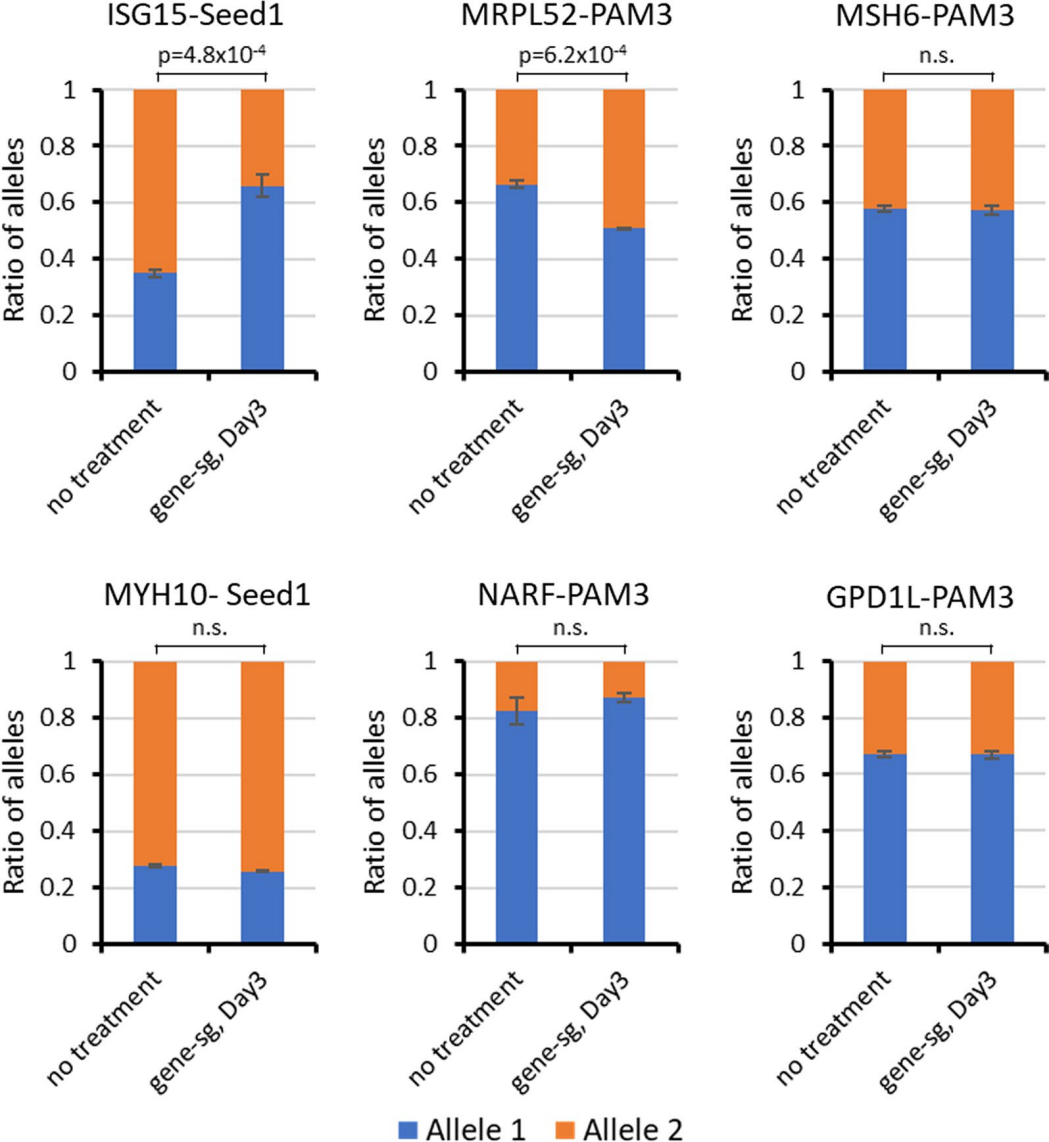
Allelic expression level analysis of target alleles before and after ASM. The ratios of the allelic reads in cDNA for untransfected and transfected samples are plotted. Error bars show the SD of two independent biological repeats. *P*-values are based on two-sided t-test assuming equal variance. n.s., not significant

Next, we were interested to determine if the introduced ASM has the capacity to alter gene expression in an allele-specific manner. The genes ISG15, MSH6, MYH10, MRPL52, NARF and GPD1L contain SNPs in an exon allowing to discriminate the expression of both alleles. Therefore, the allelic expression ratios were analyzed for these genes with and without introduction of ASM. RNA was extracted from the cells sorted on day 3 post transfection and from untreated cells and cDNA was generated. Pairs of primers were designed to amplify the corresponding exonic regions containing the SNP followed by library generation and NGS. For each gene, the number of reads per allele was extracted from the sequencing data. The ratio of the reads of each allele was calculated for the untransfected and transfected samples. A change in the ratios between untransfected and the transfected samples indicates an alteration in the expression levels of the alleles of this gene. Among the genes tested, RNA of the transfected samples ISG15-Seed2 and MRPL52-PAM3 displayed a clear shift in the ratio of alleles after ASM when compared to the parental allele ratio (Fig. 7). The ratio of allele reads from ISG15-Seed2 in untransfected samples was 35:65, while after ASM establishment, a ratio of 66:34 was observed, corresponding to a 3.6-fold change of the expression ratio (*p* value 4.8 × 10^–4^, based on a two-sided t-test assuming equal variance). The allelic reads ratio of MRPL52 before and after transfection was 66:34 and 49:51, respectively, corresponding to a twofold change (*p*-value 6.2 × 10^–4^, based on a two-sided t-test assuming equal variance). The other studied genes did not show changes in the allelic read ratios.

## Discussion

Previous work has demonstrated the ability of sgRNA/Cas9 complexes to interact with their genomic target sites in a locus specific manner if SNPs are present in critical parts of the target region and in such cases allele-specific genome editing could be achieved [37]. In this work, we aimed to apply catalytically inactivated Cas9 (dCas9) [12] to develop and characterize highly specific epigenome editing systems which are able to deliver DNA methylation specifically to one allele of the target region and establish ASM. In these experiments, the allele-specific binding of the sgRNA/dCas9 complex should be used to target a DNMT and control its activity. This adds potential problems to the experimental setting when compared with allele specific genome editing, because the DNMTs bind to DNA themselves, which could lead to untargeted methylation activity of the DNMT parts of the EpiEditors totally independent of the sgRNA/dCas9 part. We used an improved DNMT3A/3L as catalytic part that was designed to exhibit reduced non-specific activity [14]. The sgRNA/dCas9 and DNMT3A/3L parts were connected by a SunTag [15] for additional signal amplification and to facilitate DNMT3A/3L dimerization which is necessary for its catalytic activity. The design aimed to achieve allelic discrimination by targeting a heterozygous SNP either within the seed region of the sgRNA or in the PAM region of dCas9. In general, our data show that efficient ASM was achieved in many cases, though it did not work at some loci and with some of the sgRNAs. In the following, the performance of the newly designed EpiEditors for ASM will be discussed considering the most important properties: efficiency of targeted DNA methylation, specificity and stability of the ASM, and effects of ASM on allele-specific gene expression.

### Efficiency of targeted DNA methylation

Average DNA methylation levels varied across the targets irrespective of the allele targeting strategy. However, most successful targets reached an average DNA methylation above 50% at the selected CpG sites indicating high efficiency of the targeted DNA methylation. DNA methylation variation among the targets could be explained either by the sgRNA properties or the inherent epigenetic regulation at the region. The sgRNA efficiency depends on the binding capacity of the sgRNA to the target region. As the allele discriminating SNP had to be positioned in the PAM or sgRNA seed region, some sgRNA had to be used in our study although their efficiency predicted by CRIPOR (http://crispor.tefor.net/crispor.py) [38] and CCTop (https://cctop.cos.uni-heidelberg.de/) [39, 40] was only medium. Among them, the RALB and RAF1 loci targeting sgRNAs had the poorest predicted efficiencies and they indeed did not lead to efficient DNA methylation. In addition, a condensed chromatin environment of the target region could have an impact on the efficiency of epigenome editing by restricting the access to the region. In this respect, the results of previous untargeted DNA methylation experiments of CpG islands in HEK293 cells were inspected [23]. Evidently, the promoter regions of TTC41P and TYK2 remained unmethylated in the untargeted DNA methylation study and ASM could also not be established at these loci in the current work, suggesting that the local chromatin structure makes it inaccessible for DNA methylation.

### Specificity of ASM

**Fig. 8:**
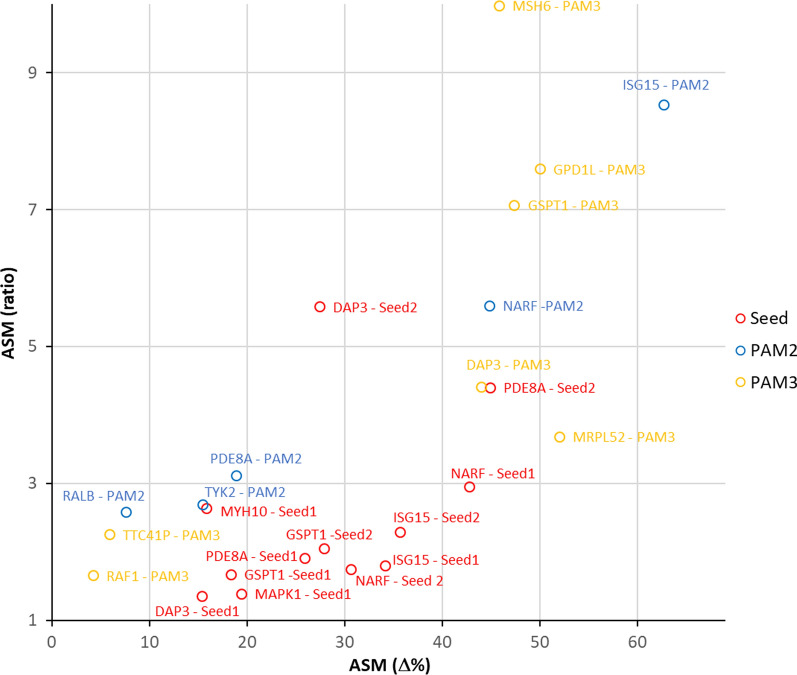
Compilation of the efficiency of ASM in all 24 experiments conducted in this study. Experiments with the SNP in the seed region of the sgRNA, at the PAM2, or at the PAM3 site are shown in red, blue and yellow, respectively. “ASM (Δ%)” and “ASM (ratio)” refer to the difference and ratio of the increase in DNA methylation at the on- and off-target alleles

For the evaluation of the success of ASM, two parameters are critical: (1) the overall level of ASM determined by the difference of the DNA methylation gain in the on- and off-target allele, and (2) the ratio of DNA methylation gain at the on- and off-target allele. In a scatter plot with ASM difference on the x- and ASM ratio on the y-axis (Fig. 8), the most successful experiments are found in the upper right corner (quadrant I), while most unsuccessful ones appear in the lower left corner (quadrant III). Inspection of the data shown in Fig. 8 indicates that the most successful ASM experiments had the SNP placed in the PAM region. This global observation agrees with the direct comparison of ASM levels achieved at loci which could be addressed in different ways (Fig. 5). Allele discrimination via a SNP in the sgRNA seed region can also be efficient, but in our data set, the best results obtained with seed-SNPs were inferior to the best PAM-based results, and the chances of inducing a strong ASM were also higher if the SNP was present in the PAM site.

In ASM, an undesired DNA methylation at the off-target allele can result from two scenarios, either binding of the sgRNA/dCas9 complex to the off-target allele, or untargeted activity of the EpiEditor enzymatic part. In our study, the residual increase in DNA methylation levels of many off-target alleles was comparable to methylation changes in samples treated with scrambled sgRNA and to the off-target locus methylation observed at the VEGFA locus (see for example DAP3-Seed2, ISG15-PAM2, or MSH6-PAM3). These observations clearly indicate that the off-target DNA methylation was caused by an untargeted activity of DNMT3A/3L in these experiments. Of note, the untargeted activity still depends on the accessibility of the genomic loci, hence it is not expected to be equal at all loci. The problem of untargeted activity of the enzymatic parts of EpiEditors had been noted in previous studies [14, 30, 31] and the untargeted activity of the DNMT3A/3L construct used here had been reduced markedly by enzyme engineering [14].

However, in some other experiments, like ISG15-Seed2, NARF-Seed1, MYH10-Seed1, MRPL52-PAM3 or DAP3-Seed2, the DNA methylation levels of the off-target allele were higher than the DNA methylation observed after treatment with scrambled sgRNA or at the off-target VEGFA locus. In these cases, an undesired binding of the sgRNA/dCas9 complex to the off-target allele must have occurred, indicating that the allele discrimination of the EpiEditors was not ideal in in these experiments, in addition to methylation background caused by untargeted activity of the DNMT3A/3L. The conclusion that off-target allele methylation is caused by binding of the sgRNA/dCas9 complex to the off-target allele in these cases is further validated by the observation that the characteristic sgRNA/dCas9 footprint is clearly visible in the off-target allele DNA methylation patterns.

There are several factors that were reported to affect the efficiency and specificity of sgRNA/dCas9 complexes: (1) SNPs in the PAM region leading to G to Y changes were shown to allow best discrimination of dCas9 binding [27]. (2) Potential formation of quadruplex DNA in the sgRNA binding region has been reported to lower the efficiency of sgRNA/dCas9 binding [41]. (3) The presence of multiple PAM sites in the seed region could reduce efficiency [42]. It is unclear if these factors act in concert and if they are dependent on further properties of the sgRNA and PAM site. We inspected the 8 most unsuccessful experiments in our data set and observed that all of them contained one or even several of these potentially negative features: TTC41P-PAM3, MAPK1-Seed1, MYH10-Seed1, RALB-PAM2, TYK2-PAM2 and DAP3-Seed1 contain multiple PAM sites and quadruplex binding sites either in the sense or antisense strand of the sgRNA binding site. RAF1-PAM3 carries a G-to-A exchange in the PAM site and it contains a quadruplex forming region in the antisense strand. GSPT1-Seed1 contains multiple PAM sites in the sgRNA binding site (in this case a G5 sequence). We conclude that avoiding these features as far as possible is advisable for the design of an ASM construct.

### Stability of ASM

For any clinical application, a durable reprogramming of the epigenome is desirable. Though this was not the main aim of our study, we analyzed the stability of the introduced ASM over 11 days. Flow cytometry revealed that the protein levels of the three fluorophores that are co-expressed with the EpiEditor declined strongly at day 8. As these fluorophores are expected to be more stable than the sgRNAs and the dCas9-10× SunTag and scFv-DNMT3A fusion proteins, one can assume that targeted DNA methylation by the EpiEditors had stopped around day 8 post transfection the latest. The DNA methylation data demonstrated at all target regions a maximum of DNA methylation on day 3, which was expected as the highest expression of the EpiEditing components was observed on day 3 after transient transfection. Afterwards, DNA methylation levels declined in most cases. However, our data demonstrated that high ASM levels (~ 90%) were retained at some of the targets up to day 11 indicating autonomous propagation of the ASM in cells at least for some time. The variable stability of ASM could arise from the local chromatin nature of the loci [23, 36]. In this respect, it is also noteworthy that HEK293 is a rapidly dividing cell line which accelerates the passive loss of DNA methylation. Hence, it is plausible to assume that ASM could be more stable in non-dividing or slowly dividing cells, like neurons. Future work may employ delivery of more than one EpiEditor which has been shown to improve durability of epigenome reprogramming [43–47]. For example, using DNMT epigenome editing combined with recruitment of KRAB would be a good option in increase the chances for durable changes of the chromatin state [43, 47].

### Allele-specific expression changes

Due to the presence of an additional SNP in the exonic regions of the corresponding genes, we could investigate allelic expression ratios of six target loci after successful establishment of ASM. Among them, ISG15-Seed2 and MRPL52-PAM3 showed a noticeable variation in the allelic read ratios. This result is an indicator of varied transcription rates at each allele before and after EpiEditing, although we note a potential PCR bias as possible pitfall of this indirect analysis approach. We conclude that allele-specific epigenome editing can be applied to regulate the allele-specific gene expression. In other cases where allelic expression ratios did not change despite of the successful establishment of ASM, the region of ASM may not have covered the relevant gene regulatory elements, which would explain a lack of direct effects on allelic expression levels. This highlights a general difficulty in the epigenome editing technology, where it needs to be ensured that the epigenome reprogramming occurs at genomic sites relevant for the expression of the target gene.

Other experimental approaches have been used for allele-specific silencing of dominant mutant alleles in diseases including short interfering RNA (siRNA) [48] and allele-specific silencing of poly-Q containing alleles was achieved by miRNA targeting [49]. Antisense oligonucleotides (ASOs) were used for allele-specific targeting of the Huntingtin gene [50, 51], and rhodopsin P23H gene in retinitis pigmentosa, where ASO-mediated silencing led to long-term effects in rodent models [52]. However, one limiting factor of ASO is the protein level of RNaseH1 needed for the degradation of the mutant mRNA. Moreover, miRNA and ASO-based approaches are transient by nature, and require regular application of the reagents. In contrast to this, genome and epigenome editing has the potential to cause durable effects. For example, a permanent inactivation of Huntington's disease mutation had been achieved by allele-specific genome editing using sgRNA/Cas9 [53], but this treatment is based on the generation of mutations. In contrast, epigenome editing has the advantage that no DNA mutations are introduced. Indeed, allele-specific transcriptional repression of the mutant Huntingtin gene had been achieved by zinc finger or TALE fused KRAB domain and it was shown to result in long term reduction of the expression of the disease allele at least in one example [54, 55]. Our study demonstrates that sgRNA/dCas9 complexes, which mediate flexible targeted epigenome editing based on Watson/Crick base pairing between the sgRNA and its target locus DNA, can be used in a similar way for introducing ASM in personalized medicine approaches.

### Applicability of the ASM approach

The general applicability of the ASM approach depends on the availability of a heterozygous SNP combined with a potential sgRNA/dCas9 binding site within the gene regulatory elements of the target gene. It has been found that HEK293 cells contain 3.6 million heterozygous SNPs [22]. Assuming that the gene regulatory promoter region is 500 bp downstream and 50 bp upstream of a transcriptional start site (TSS), 39% of all human TSS were found to contain a SNP in HEK293 cells. Using a seed region of 7 bps, 64% of these SNPs carry a suitable PAM/sgRNA binding site nearby. Hence, overall 25% of all TSSs can be directly targeted by the ASM approach developed here in HEK293 cells. However, the actual chance to target a given medically relevant gene will be even higher, because the targeting probability increases with the size of the gene regulator elements. The gene regulatory regions of most medically relevant genes are well-studied, and the total sizes of the functional promoter and enhancer elements usually are much larger than the 550 bps per TSS used in our estimation here.

## Conclusions

In this study, we demonstrated allele-specific deposition of DNA methylation in the promoter region of several genes using sgRNA/dCas9 complexes. While we have used HEK293 as model cell line to establish ASM, our results most likely will be transferable to other cell lines and primary cells, provided that suitable targeting systems are available. Depending on the target locus and method of allele discrimination, we show high specificity and efficiency of ASM which was stable over several days in some cases. Moreover, ASM was shown to change the allelic ratio of gene expression at the target loci indicating that this technology could potentially be employed in the clinic to control the progression of diseases caused by the expression of a dominant disease allele.

## Methods

### sgRNA design and cloning

A single sgRNA expression vector was produced by replacement of a part of the backbone of the pMulti-sgRNA-LacZ-DsRed vector (a gift from Yujie Sun, Addgene plasmid #99914) [56] with the sgRNA expression cassette from the AIO-mCherry vector (a gift from Steve Jackson, Addgene plasmid #74120) [57]. For targets with SNP in the PAM region, a genomic sequence of 20 nt upstream of the PAM was selected as sgRNA binding site. For targets with SNP in the sgRNA seed region, a 20 nt sequence upstream of the nearest PAM (containing the SNP) was used. The selected sgRNAs were evaluated for the efficiency and potential off-targets using CRIPOR (http://crispor.tefor.net/crispor.py) [38] and CCTop (https://cctop.cos.uni-heidelberg.de/) [39, 40]. The potential formation of quadruplex DNA in the sgRNA region was analyzed by QGRS Mapper [58]. All target regions and sgRNAs are schematically shown in Additional file 1. As a negative control, a scrambled sgRNA with following sequence GAACAGTCGCGTTTGCGACT was used which does not have a target in the human genome [14, 29].

For cloning of sgRNA expression vectors, the top and bottom strand oligonucleotides designed for each target region were mixed (5 µM) in annealing buffer (NEB buffer2: 50 mM NaCl,10 mM Tris/HCl pH 7.9, 10 mM MgCl_2_ and 1 mM DTT). All sgRNA sequences are listed in Additional file 3: Table S1. Annealing was carried out in a thermocycler using the program: heat to 90 °C, followed by cooling to 20 °C with 1 °C per minute. Annealed oligos were cloned into the single sgRNA expression vector by Golden Gate Assembly. Kanamycin resistant positive clones were sequenced to confirm the sgRNA expression cassette. Afterwards, four to six sg-RNAs were cloned into each multi-sgRNA vector. For this, the region covering the U6 promoter, inserted target guide and the gRNA scaffold from the single sgRNA vector was amplified using the primers provided in the Additional file 3: Table S2. The primers were designed with suitable overhangs for ligation after BbsI digestion. The amplified regions were cloned into the pMulti-sgRNA-LacZ-DsRed vector (a gift from Yujie Sun, Addgene plasmid # 99914) [56] by Golden Gate Assembly.

### Cell culture and transfection

HEK293 cells (RRID: CVCL_0045) were cultivated in Dulbecco’s modified Eagle’s Medium (D5671, Sigma) supplemented with 10% Fetal Bovine Serum (F7524, Sigma), L-Glutamine (G7513, Sigma) and 1× PenStrep (Sigma, P0781). For subculture and harvest, the cells were washed with PBS (D802, Sigma) and treated with trypsin (T3924, Sigma). For transfections, 1.4 million cells were seeded in 100 mm dish the day prior to transfection. The cells were transfected with a mixture of three constructs: the dCas9-10× SunTag (Addgene #174141) [14] (6 µg), scFv-DNMT3A(R887E)-DNMT3L (Addgene #154141) [14] (3 µg) and the multi-sgRNA expression vector (0.5 g). Every plasmid additionally expressed a fluorescent marker, namely the plasmids encoding dCas9-10× SunTag BFP, scFv-DNMT3A/3L sfGFP, and sgRNA DsRed, respectively. FuGENE® HD was used as the transfection reagent according to the manufacturer’s guideline. The growth medium was replaced the next day. Three days after transfection, the cells were trypsinised and passed through a pre-separation filter of 30 µm (Cat no: 130-041-407, Miltenyi Biotec). Filtered cells were sorted by SH800S Cell Sorter (Sony Biotechnology) to obtain the triple-positive population, using the gating strategy shown in Additional file 2: Fig. S8. About 0.5 million triple-positive cells were obtained for every sample and used for further experiments.

### DNA methylation analysis

Genomic DNA was extracted using QIAmp DNA Mini Kit (Qiagen). A total of 500 ng genomic DNA was subjected to overnight digestion with EcoRV, a non-cutter for all the target amplicons. Zymo EZ DNA Methylation-Lightning Kit (D5030-E) was used for bisulfite conversion. The library for NGS was prepared by two consecutive PCR reactions [59]. Firstly, bisulfite converted genomic DNA of each sample was amplified with target gene specific primers provided in Additional file 3: Table S3. The gene specific optimized amount of a product from the first PCR was used as a template for the second PCR to add the Illumina TruSeq sequencing adapters (Additional file 3: Table S4). Final products were quantified, pooled in equimolar amounts and purified using SPRIselect beads (Beckman Coulter). Ready-to-use pools of libraries were sequenced on NovaSeq 6000 using a PE250 flow cell (Novogene). NGS data were obtained in the form of FASTQ files, which were processed on the local instance of Galaxy server as described earlier [60] with some modifications. Briefly, an adaptor and low-quality trimming was conducted using Trim Galore! (https://github.com/FelixKrueger/TrimGalore). The two associated paired reads were merged using PEAR [61]. Experiment specific combinations of Illumina indices and barcodes were used to extract reads for the individual experiments from the pool of reads. Reads corresponding to different alleles of the same target gene were identified based on the presence of the SNP. Two files of reads corresponding to alleles were generated and their DNA methylation level was analyzed independently. First, reads were mapped against a reference sequence of the target region using bwameth [62] and then DNA methylation of individual CpG sites was computed using MethylDackel (https://github.com/dpryan79/MethylDackel). Final visualization and statistics were prepared using Microsoft Excel.

### RNA isolation and expression analysis

RNA was extracted from sorted cells using Qiagen RNeasy extraction kit (Cat. No. 74034). 500 ng of the purified RNA was used for the cDNA synthesis with the Applied Biosystems- High-Capacity cDNA Reverse Transcription Kit (Cat No 4368814). The expression analysis was performed for the genes with additional SNPs in one of the exons. For each gene, a pair of primers was designed to amplify the exonic regions with SNP. Library preparation for this region was performed in by two step PCR. In the first PCR, cDNA was amplified with the target specific primers provided in Additional file 3: Table S5 using Hot start Taq Polymerase (95 °C for 15 min, 20 cycles of 94 °C—30s, T_A_—30 s and 72 °C for 60 s, and 72 °C for 10 min). Second PCR for library generation was performed using the undiluted product from the previous PCR amplified with Illumina library specific primers provided in Additional file 3: Table S4. Q5 polymerase was used for amplification (98 °C for 30 s, 15 cycles of 98 °C for 10s and 72 for 40 s, and 72 °C for 2 min). The allelic expression studies were conducted in two independent biological replicates.

### Estimation of the applicability of the ASM approach

A list of all heterozygous SNPs in HEK293 cells was taken from http://hek293genome.org/v2/ [22]. The combined list of all human TSS was taken from refTSS v3.1 (https://reftss.riken.jp/datafiles/3.1/) [63].

### Supplementary Information

**Additional file 1**. Schematic images showing the target loci, allele discrimination strategy and sgRNA binding sites within the analyzed DNA region.

**Additional file 2: Fig. S1.** Overview of the workflow for ASM applied here. **Fig. S2.** DNA methylation observed after targeting a SNP in the seed region of the sgRNA for additional experiments with weak or no ASM. **Fig. S3.** DNA methylation profiles observed at the VEGFA locus as an off-target locus control. **Fig. S4.** DNA methylation observed after targeting a SNP at the second PAM position for additional experiments with weak or no ASM. **Fig. S5.** DNA methylation profiles observed after targeting a SNP at the third PAM position for experiments with weak or no ASM. **Fig. S6.** Stability of BFP, sfGFP and DsRed observed in the experiments investigating the stability of ASM. **Fig. S7.** DNA methylation profiles observed in the experiments investigating the stability of ASM. **Fig. S8.** Gating strategy used for the sorting of cells containing all three plasmids which encode the components of the EpiEditing complex and co-express BFP, sfGFP and DsRed.

**Additional file 3: Table S1.** List of oligodeoxynucleotide used in sgRNA cloning. **Table S2.** List of primers used for multi-sgRNA cloning. **Table S3.** List of target specific primers used for amplifying bisulfite treated gDNA samples. **Table S4.** List of Illumina specific primers used in the second PCR reaction. **Table S5.** List of primers used for transcript amplification from cDNA.

## Data Availability

Primary sequencing data are published in DARUS (https://doi.org/10.18419/darus-3581). All analyzed data are included in this published article and its supplementary information files.
