# Supplementary material for: Development of super-specific epigenome editing by targeted allele-specific DNA methylation
Source: Epigenetics Chromatin. 2023 Oct 21;16:41. doi: 10.1186/s13072-023-00515-5 (PMC10589950; doi:10.1186/s13072-023-00515-5)
Supplement: Supplementary file 1 — Additional file 1. Schematic images showing the target loci, allele discrimination strategy and sgRNA binding sites within the analyzed DNA region. [file 13072_2023_515_MOESM1_ESM.pdf]

Development of super-specific epigenome editing by targeted allele-specific DNA methylation

Additional file 1

Schematic images showing the target loci and allele discrimination strategy.

The images in the top panel represent the positions of the sgRNA binding site (red bar), the genomic region analyzed by NGS (blue bar) and the TSS of the associated gene starting at position +1 (green arrow). The numbers indicate the distance in bps from the TSS. Some of the features are not drawn to scale.

The image in the lower panel indicate the strategy for discrimination of alleles with SNP in the PAM site or sgRNA seed region. The sequence of sgRNA for each target region and the binding site of the sgRNA in the targeted allele is highlighted. The regions are color coded with the sgRNA sequence in red, the binding site of the sgRNA in violet, PAM site in green, and the SNP is indicated with a red arrow and written in bold text. The designed sgRNA binds to the targeted allele mentioned in each figure. In the untargeted allele, the presence of the SNP in the PAM or sgRNA seed region, disfavors the binding of the sgRNA/dCas9 complex.

On the last page of this file, the sgRNA binding sites within the analyzed target regions and the corresponding the CpG site numbers are provided.

DAP3-PAM3

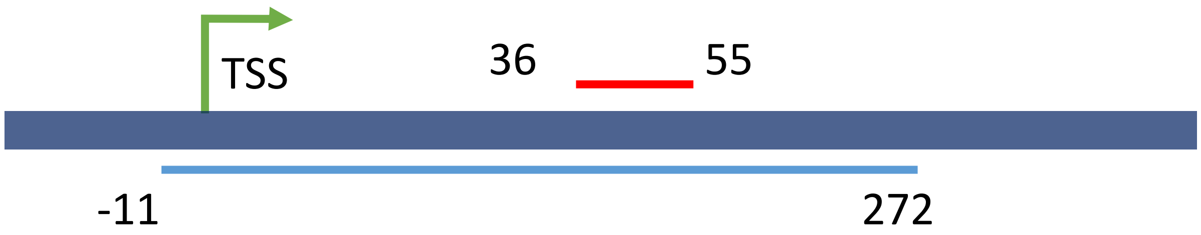

Region	Genome coordinates (hg19)
**SNP**	chr1:155658940-155658940
**sgRNA**	chr1:155658918-155658937 (+)
**NGS analysis region**	chr1:155658871-155659154

DAP3-PAM3 sgRNA designed to target Allele 1

sgRNA

CCGCGGTTGTCTCCAGGCCA

Allele 1

```
                    CCGCGGTTGTCTCCAGGCCA
5‘CGCTTTGGAGCCGGCCCCAGGCAGCGTGTGTCGGTCGCCTAGGCTGGAGAACTAGTCCTCGAC3‘
3‘GCGAAACCTCGGCCGGGGTCCGTCGCACACAGCCAGCGGATCCGACCTCTTGATCAGGAGCTG5‘
```

Allele 2

```
5‘CGCTTTGGAGCCGGCCCCAGGCAGCGTGTGTCGGTCGCCTAGTCTGGAGAACTAGTCCTCGAC3‘
3‘GCGAAACCTCGGCCGGGGTCCGTCGCACACAGCCAGCGGATCAGACCTCTTGATCAGGAGCTG5‘
```

DAP3-Seed1

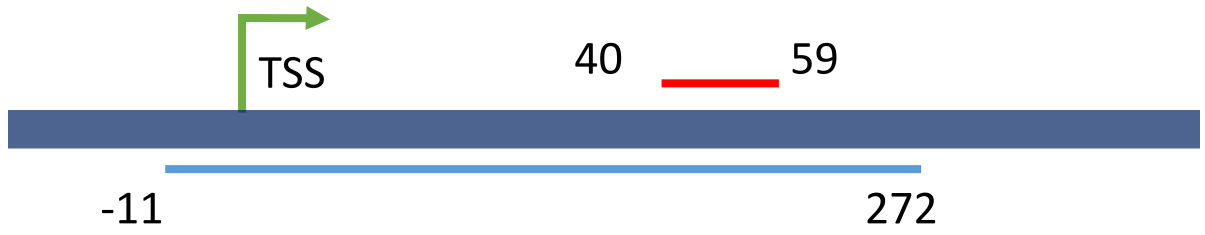

Region	Genome coordinates (hg19)
SNP	chr1:155658940-155658940
sgRNA	chr1:155658922-155658941 (+)
NGS analysis region	chr1:155658871-155659154

DAP3-Seed1 sgRNA designed to target Allele 1

sgRNA

CGTGTGTCGGTCGCCTAGTC

Allele 1

CGTGTGTCGGTCGCCTAGGC

5'TTGGAGCCGGCCCCAGGCAGCGTGTGTCGGTCGCCTAGGCTGGAGAACTAGTCCTCGACTCAC3'

3'AACCTCGGCCGGGGTCCGTCGCACACAGCCAGCGGATCCGACCTCTTGATCAGGAGCTGAGTG5'

Allele 2

5'TTGGAGCCGGCCCCAGGCAGCGTGTGTCGGTCGCCTAG**T**CTGGAGAACTAGTCCTCGACTCAC'

3'AACCTCGGCCGGGGTCCGTCGCACACAGCCAGCGGATC**A**GACCTCTTGATCAGGAGCTGAGTG5'

DAP3-Seed2

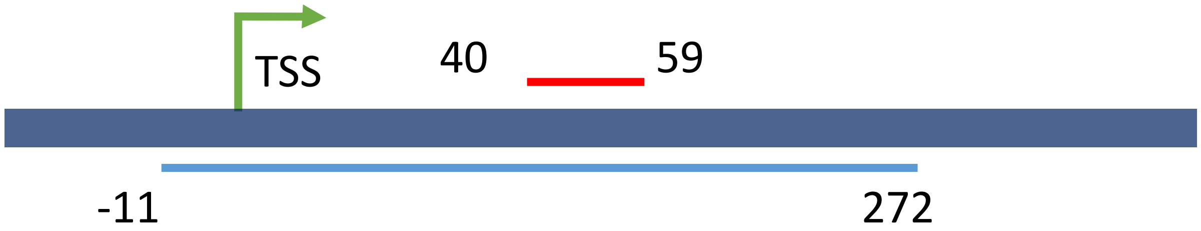

Region	Genome coordinates (hg19)
**SNP**	chr1:155658940-155658940
**sgRNA**	chr1:155658922-155658941 (+)
**NGS analysis region**	chr1:155658871-155659154

DAP3-Seed2 sgRNA designed to target Allele 2

sgRNA

CGTGTGTCGGTCGCCTAGGC

Allele 1

5'TTGGAGCCGGCCCCAGGCAGCGTGTGTCGGTCGCCTAG**G**CTGGAGAACTAGTCCTCGACTCAC3'

3'AACCTCGGCCGGGGTCCGTCGCACACAGCCAGCGGATC**C**GACCTCTTGATCAGGAGCTGAGTG5'

Allele 2

CGTGTGTCGGTCGCCTAGTC

5'TTGGAGCCGGCCCCAGGCAGCGTGTGTCGGTCGCCTAGTCTGGAGAACTAGTCCTCGACTCAC'

3'AACCTCGGCCGGGGTCCGTCGCACACAGCCAGCGGATCCGACCTCTTGATCAGGAGCTGAGTG5'

GPD1L-PAM3

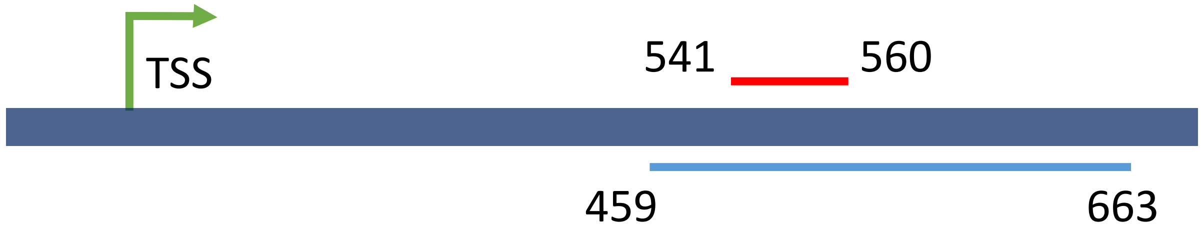

Region	Genome coordinates (hg19)
SNP	chr3:32148716-32148716
sgRNA	chr3:32148694-32148713 (+)
NGS analysis region	chr3:32148612-32148816

GPD1L-PAM3 sgRNA designed to target Allele 1

sgRNA

CTTGCTGCCCAGGTCACGGG

Allele 1

```
                    CTTGCTGCCCAGGTCACGGG
5'GCGGAGAGGGCTAAAGGGCTCTTGCTGCCCAGGTCACGGGCGGGGCATGCTCTGCCCTTCAAG3'
  |||||||||||||||||||||||||||||||||||||||||||||||||||||||||||||||
3'CGCCTCTCCCGATTTCCCGAGAACGACGGGTCCAGTGCCCGCCCCGTACGAGACGGGAAGTTC5'
```

Allele 2

```
5'GCGGAGAGGGCTAAAGGGCTCTTGCTGCCCAGGTCACGGGCGTGGCATGCTCTGCCCTTCAAG3'
  |||||||||||||||||||||||||||||||||||||||||||||||||||||||||||||||
3'CGCCTCTCCCGATTTCCCGAGAACGACGGGTCCAGTGCCCGCACCGTACGAGACGGGAAGTTC5'
```

GSPT1-PAM3

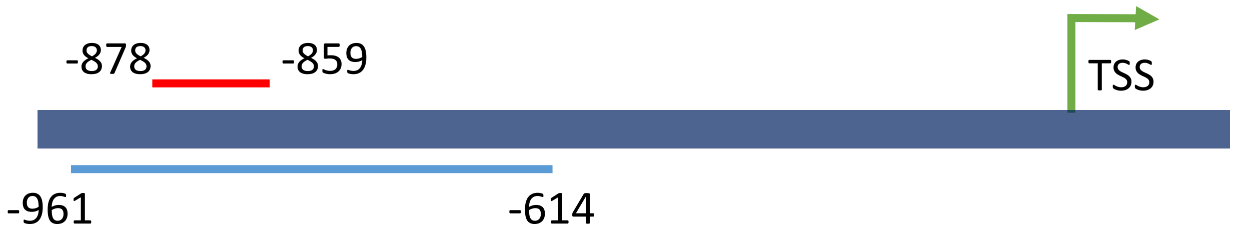

Region	Genome coordinates (hg19)
SNP	chr16:12008941-12008941
sgRNA	chr16:12008919-12008938 (+)
NGS analysis region	chr16:12008836-12009183

GSPT1-PAM3 sgRNA designed to target Allele 1

sgRNA

TCCATACGGTTCCCATCTCA

Allele 1

```
                     TCCATACGGTTCCCATCTCA
5'CGCCGCGGCCCCCACTCCGTTCCATACGGTTCCCATCTCAAGGGGGTAACTGCGGACTCCAGA3'
  ||||||||||||||||||||||||||||||||||||||||||||||||||||||||||||||
3'GCGGCGCCGGGGGTGAGGCAAGGTATGCCAAGGGTAGAGTTCCCCCATTGACGCCTGAGGTCT5'
```

Allele 2

```
5'CGCCGCGGCCCCCACTCCGTTCCATACGGTTCCCATCTCAAGTGGGTAACTGCGGACTCCAGA3'
  ||||||||||||||||||||||||||||||||||||||||||||||||||||||||||||||
3'GCGGCGCCGGGGGTGAGGCAAGGTATGCCAAGGGTAGAGTTCACCCATTGACGCCTGAGGTCT5'
```

GSPT1-Seed1

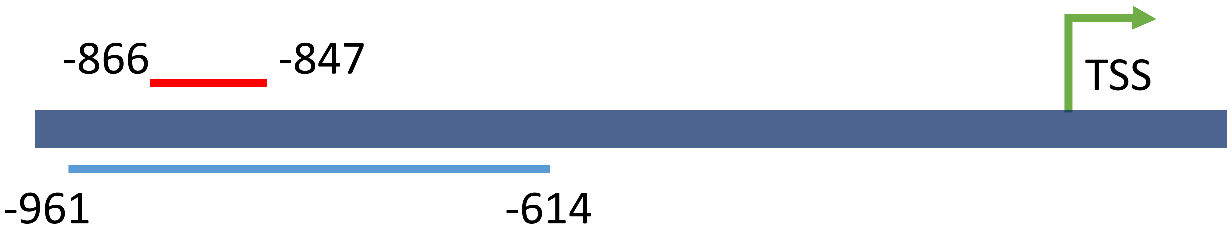

Region	Genome coordinates (hg19)
SNP	chr16:12008941-12008941
sgRNA	chr16:12008931-12008950 (+)
NGS analysis region	chr16:12008836-12009183

GSPT1-Seed1 sgRNA designed to target Allele 1

sgRNA

CCATCTCAAGGGGGTAACTG

Allele 1

```
                    CCATCTCAAGGGGGTAACTG
5'CACTCCGTTCCATACGGTTCCCATCTCAAGGGGGTAACTGCGGACTCCAGAGCAGGGCAGGGG3'
  |||||||||||||||||||||||||||||||||||||||||||||||||||||||||||||||
3'GTGAGGCAAGGTATGCCAAGGGTAGAGTTCCCCCATTGACGCCTGAGGTCTCGTCCCGTCCCC5'
```

Allele 2

```
5'CACTCCGTTCCATACGGTTCCCATCTCAAGTGGGTAACTGCGGACTCCAGAGCAGGGCAGGGG3'
  |||||||||||||||||||||||||||||||||||||||||||||||||||||||||||||||
3'GTGAGGCAAGGTATGCCAAGGGTAGAGTTCACCCATTGACGCCTGAGGTCTCGTCCCGTCCCC5'
```

GSPT1-Seed2

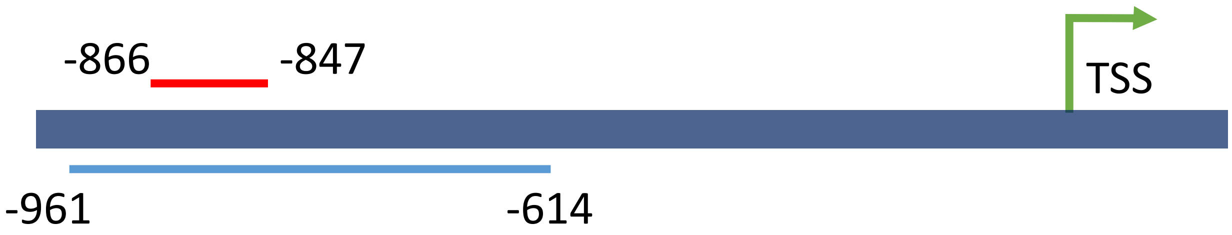

Region	Genome coordinates (hg19)
**SNP**	chr16:12008941-12008941
**sgRNA**	chr16:12008931-12008950 (+)
**NGS analysis region**	chr16:12008836-12009183

GSPT1-Seed2 sgRNA designed to target Allele 2

sgRNA

CCATCTCAAGTGGGTAACTG

Allele 1

```
5'CACTCCGTTCCATACGGTTCCCATCTCAAGGGGGTAACTGCGGACTCCAGAGCAGGGCAGGGG3'
  |||||||||||||||||||||||||||||||||||||||||||||||||||||||||||||||
3'GTGAGGCAAGGTATGCCAAGGGTAGAGTTCCCCCATTGACGCCTGAGGTCTCGTCCCGTCCCC5'
```

Allele 2

```
                    CCATCTCAAGTGGGTAACTG
5'CACTCCGTTCCATACGGTTCCCATCTCAAGTGGGTAACTGCGGACTCCAGAGCAGGGCAGGGG3'
  |||||||||||||||||||||||||||||||||||||||||||||||||||||||||||||||
3'GTGAGGCAAGGTATGCCAAGGGTAGAGTTCACCCATTGACGCCTGAGGTCTCGTCCCGTCCCC5'
```

ISG15-PAM2

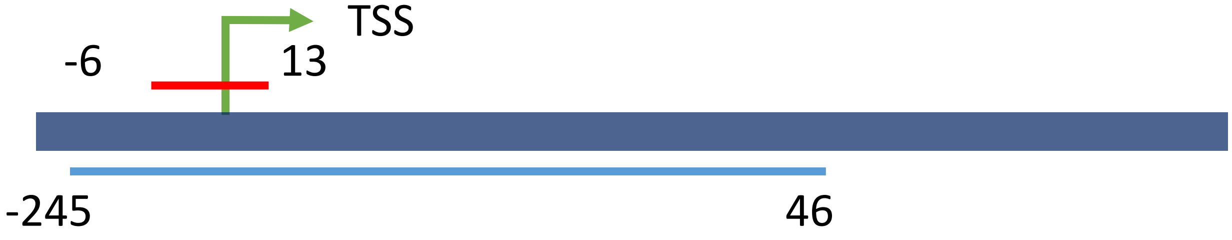

Region	Genome coordinates (hg19)
SNP	chr1:948878-948878
sgRNA	chr1:948872-948891 (-)
NGS analysis region	chr1:948633-948924

ISG15-PAM2 sgRNA designed to target Allele 1

sgRNA

TGCCTCTCAGCCGCCGGCTT

Allele 1

```
                    TGCCTCTCAGCCGCCGGCTT
5'ACTGGCAAAGATGAGTTCGCTGCCTCTCAGCCGCCGGCTTCGGCAGGCAGCACCGGCCCTATT3'
  |||||||||||||||||||||||||||||||||||||||||||||||||||||||||||||||
3'TGACCGTTTCTACTCAAGCGACGGAGAGTCGGCGGCCGAAGCCGTCCGTCGTGGCCGGGATAA5'
```

Allele 2

```
5'ACTGGCAAAGATGAGTTCGCTGCCTCTCAGCCGCCGGCTTCCGCAGGCAGCACCGGCCCTATT3'
  |||||||||||||||||||||||||||||||||||||||||||||||||||||||||||||||
3'TGACCGTTTCTACTCAAGCGACGGAGAGTCGGCGGCCGAAGGCGTCCGTCGTGGCCGGGATAA5'
```

ISG15-Seed1

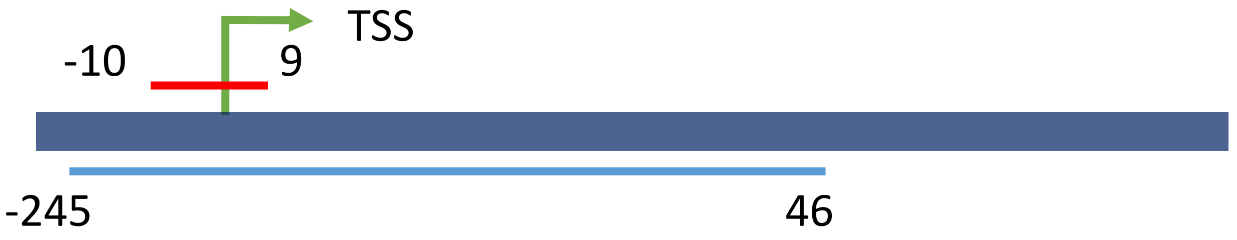

Region	Genome coordinates (hg19)
SNP	chr1:948878-948878
sgRNA	chr1:948868-948887 (-)
NGS analysis region	chr1:948633-948924

ISG15-Seed1 sgRNA designed to target Allele 1

sgRNA

TCTCAGCCGCCGGCTTCGGC

Allele 1

```
                     TCTCAGCCGCCGGCTTCGGC
5'GCAAAGATGAGTTCGCTGCCTCTCAGCCGCCGGCTTCGGCAGGCAGCACCGGCCCTATTATTA3'
  ||||||||||||||||||||||||||||||||||||||||||||||||||||||||||||||||
3'CGTTTCTACTCAAGCGACGGAGAGTCGGCGGCCGAAGCCGTCCGTCGTGGCCGGGATAATAAT'
```

Allele 2

```
5'GCAAAGATGAGTTCGCTGCCTCTCAGCCGCCGGCTTCCGCAGGCAGCACCGGCCCTATTATTA3'
  ||||||||||||||||||||||||||||||||||||||||||||||||||||||||||||||||
3'CGTTTCTACTCAAGCGACGGAGAGTCGGCGGCCGAAGGCGTCCGTCGTGGCCGGGATAATAAT'
```

ISG15-Seed2

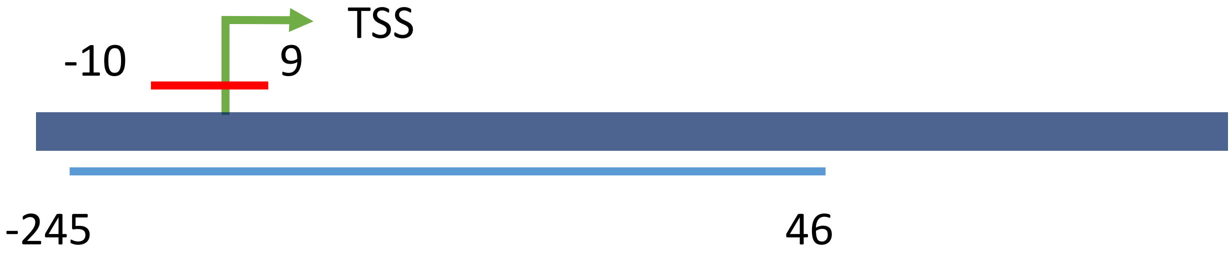

Region	Genome coordinates (hg19)
SNP	chr1:948878-948878
sgRNA	chr1:948868-948887 (-)
NGS analysis region	chr1:948633-948924

ISG15-Seed2 sgRNA designed to target Allele 2

sgRNA

TCTCAGCCGCCGGCTTCCGC

Allele 1

```
5'GCAAAGATGAGTTCGCTGCCTCTCAGCCGCCGGCTTCGGCAGGCAGCACCGGCCCTATTATTA3'
  |||||||||||||||||||||||||||||||||||||||||||||||||||||||||||||||
3'CGTTTCTACTCAAGCGACGGAGAGTCGGCGGCCGAAGCCGTCCGTCGTGGCCGGGATAATAAT5'
```

Allele 2

```
                      TCTCAGCCGCCGGCTTCCGC
5'GCAAAGATGAGTTCGCTGCCTCTCAGCCGCCGGCTTCCGCAGGCAGCACCGGCCCTATTATTA3'
  |||||||||||||||||||||||||||||||||||||||||||||||||||||||||||||||
3'CGTTTCTACTCAAGCGACGGAGAGTCGGCGGCCGAAGGCGTCCGTCGTGGCCGGGATAATAAT5'
```

MAPK1-Seed1

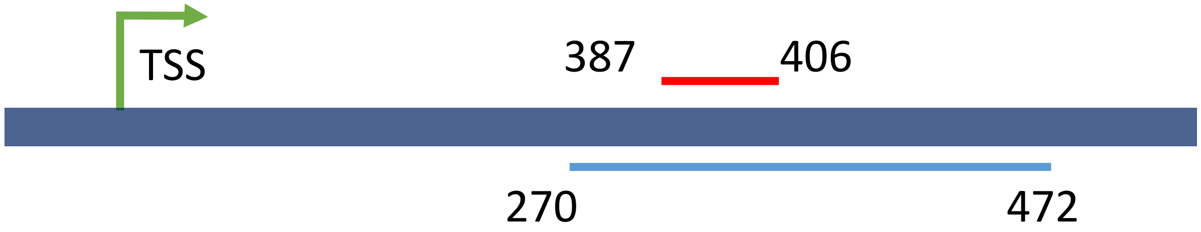

Region	Genome coordinates (hg19)
SNP	chr22:22222320-22222320
sgRNA	chr22:22222318-22222337 (-)
NGS analysis region	chr22:22222201-22222403

MAPK1-Seed1 sgRNA designed to target Allele 1

sgRNA

GCTGCGGCGCTGCCGGGATA

Allele 1

```
                    GCTGCGGCGCTGCCGGGATA
5'ACAGATTCGCAGAGGCCTGAGCTGCGGCGCTGCCGGGATATGGCGCGTCGTCGGGCCTCAAGC3'
  |||||||||||||||||||||||||||||||||||||||||||||||||||||||||||||||
3'TGTCTAAGCGTCTCCGGACTCGACGCCGCGACGGCCCTATACCGCGCAGCAGCCCGGAGTTCG5'
```

Allele 2

```
5'ACAGATTCGCAGAGGCCTGAGCTGCGGCGCTGCCGGGCTATGGCGCGTCGTCGGGCCTCAAGC3'
  |||||||||||||||||||||||||||||||||||||||||||||||||||||||||||||||
3'TGTCTAAGCGTCTCCGGACTCGACGCCGCGACGGCCCGATACCGCGCAGCAGCCCGGAGTTCG5'
```

MRPL52-PAM3

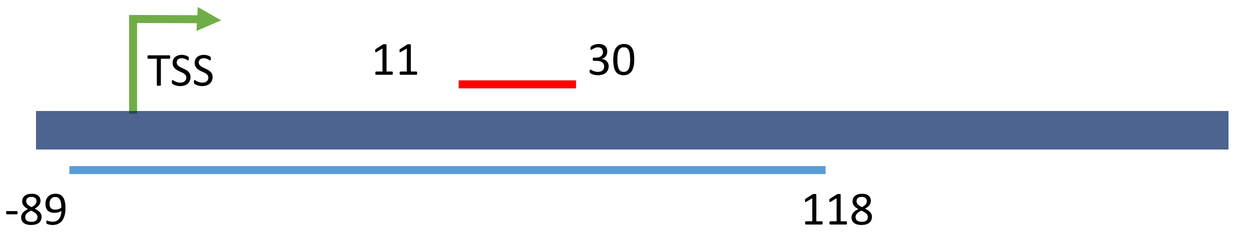

Region	Genome coordinates (hg19)
SNP	chr14:23299135-23299135
sgRNA	chr14:23299113-23299132 (+)
NGS analysis region	chr14:23299013-23299220

MRPL52-PAM3 sgRNA designed to target Allele 1

sgRNA

CTGCTCAGCATGGCTGCTTT

Allele 1

```
                    CTGCTCAGCATGGCTGCTTT
5'TCCGGCTACCCCGGCTACTCCTGCTCAGCATGGCTGCTTTAGGGACTGTTCTCTTCAGTGAGT3'
3'AGGCCGATGGGGCCGATGAGGACGAGTCGTACCGACGAAATCCCTGACAAGAGAAGTCACTCA5'
```

Allele 2

```
5'TCCGGCTACCCCGGCTACTCCTGCTCAGCATGGCTGCTTTAGTGACTGTTCTCTTCAGTGAGT3'
3'AGGCCGATGGGGCCGATGAGGACGAGTCGTACCGACGAAATCACTGACAAGAGAAGTCACTCA5'
```

MSH6-PAM3

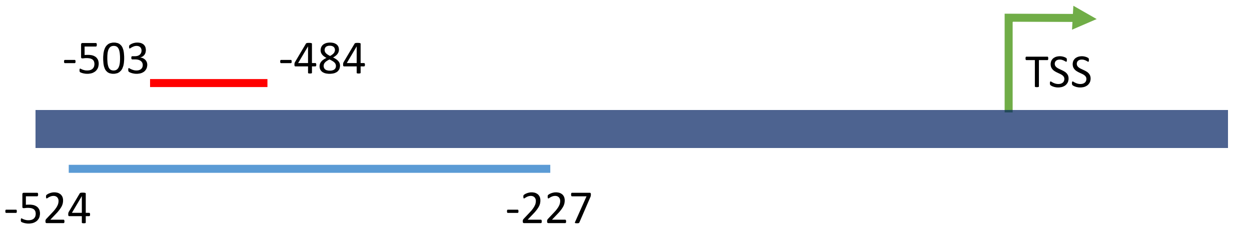

Region	Genome coordinates (hg19)
**SNP**	chr2:48009816-48009816
**sgRNA**	chr2:48009794-48009813 (+)
**NGS analysis region**	chr2:48009773-48010070

MSH6-PAM3 sgRNA designed to target Allele 1

sgRNA

AAGTTTGGTCCCTTTCGCTC

Allele 1

```
                           AAGTTTGGTCCCTTTCGCTC
5'TGAAGGTGAACTGCTGACTAAAGTTTGGTCCCTTTCGCTCCGGCTCCTTGCGAAAATGCTCTA5'
  |||||||||||||||||||||||||||||||||||||||||||||||||||||||||||||||
3'ACTTCCACTTGACGACTGATTTCAAACCAGGGAAAGCGAGGCCGAGGAACGCTTTTACGAGAT3'
```

Allele 2

```
5'TGAAGGTGAACTGCTGACTAAAGTTTGGTCCCTTTCGCTCCGTCTCCTTGCGAAAATGCTCTA5'
  |||||||||||||||||||||||||||||||||||||||||||||||||||||||||||||||
3'ACTTCCACTTGACGACTGATTTCAAACCAGGGAAAGCGAGGCAGAGGAACGCTTTTACGAGAT3'
```

MYH10-Seed1

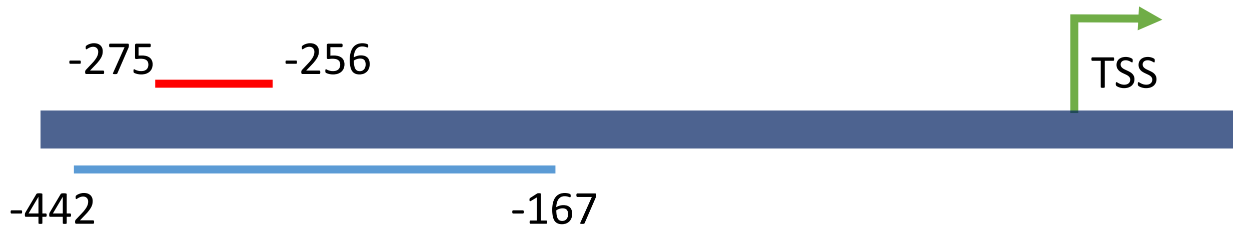

Region	Genome coordinates (hg19)
SNP	chr17:8533798-8533798
sgRNA	chr17:8533796-8533815 (-)
NGS analysis region	chr17:8533629-8533904

MYH10-Seed1 sgRNA designed to target Allele 1

sgRNA

GAGCTGTAGGGTTTGTGCTG

Allele 1

GAGCTGTAGGGTTTGTGCTG

5'GGGAAGACAGAGGGGCTTCTGAGCTGTAGGGTTTGTGCTGCGGGGACCTTAGGTTAGTTTGAG3'

3'CCCTTCTGTCTCCCCGAAGACTCGACATCCCAAACACGACGCCCCTGGAATCCAATCAAACTC5'

Allele 2

5'GGGAAGACAGAGGGGCTTCTGAGCTGTAGGGTTTGTG**T**TGCGGGGACCTTAGGTTAGTTTGAG3'

3'CCCTTCTGTCTCCCCGAAGACTCGACATCCCAAACAC**A**ACGCCCCTGGAATCCAATCAAACTC5'

NARF-PAM2

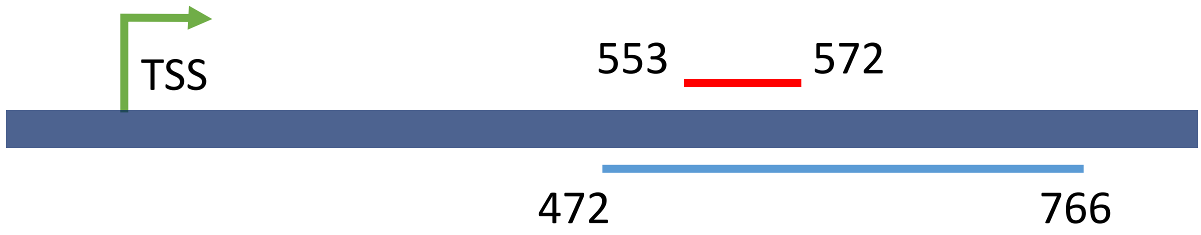

Region	Genome coordinates (hg19)
SNP	chr17:80417150-80417150
sgRNA	chr17:80417129-80417148 (+)
NGS analysis region	chr17:80417054-80417348

NARF-PAM2 sgRNA designed to target Allele 1

sgRNA

CGCCTTCCGCGGTTGTCTCC

Allele 1

```
                    CGCCTTCCGCGGTTGTCTCC
5'GGGTGACGGCTGCTGACCGGCGCCTTCCGCGGTTGTCTCCAGGCCATGGCAACAACCGCGCAG3'
3'CCCACTGCCGACGACTGGCCGCGGAAGGCGCCAACAGAGGTCCGGTACCGTTGTTGGCGCGTC5'
```

Allele 2

```
5'GGGTGACGGCTGCTGACCGGCGCCTTCCGCGGTTGTCTCCATGCCATGGCAACAACCGCGCAG3'
3'CCCACTGCCGACGACTGGCCGCGGAAGGCGCCAACAGAGGTACGGTACCGTTGTTGGCGCGTC5'
```

NARF-Seed1

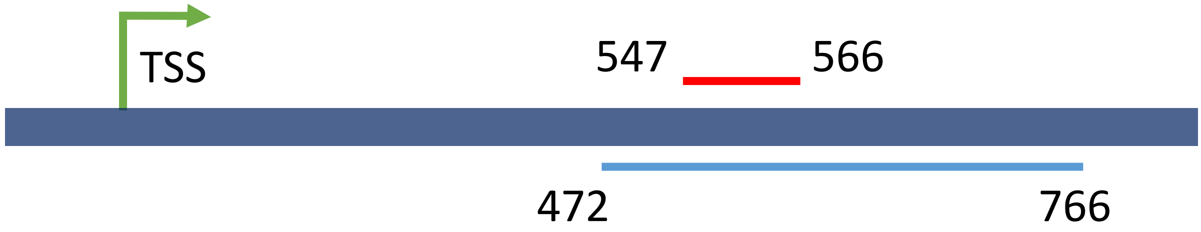

Region	Genome coordinates (hg19)
**SNP**	chr17:80417150-80417150
**sgRNA**	chr17:80417135-80417154 (+)
**NGS analysis region**	chr17:80417054-80417348

NARF-Seed1 sgRNA designed to target Allele 1

sgRNA

CCGCGGTTGTCTCCAGGCCA

Allele 1

```
                    CCGCGGTTGTCTCCAGGCCA
5'CGGCTGCTGACCGGCGCCTTCCGCGGTTGTCTCCAGGCCATGGCAACAACCGCGCAGCGCACG5'
  ||||||||||||||||||||||||||||||||||||||||||||||||||||||||||||||
3'GCCGACGACTGGCCGCGGAAGGCGCCAACAGAGGTCCGGTACCGTTGTTGGCGCGTCGCGTGC3'
```

Allele 2

```
5'CGGCTGCTGACCGGCGCCTTCCGCGGTTGTCTCCATGCCATGGCAACAACCGCGCAGCGCACG5'
  ||||||||||||||||||||||||||||||||||||||||||||||||||||||||||||||
3'GCCGACGACTGGCCGCGGAAGGCGCCAACAGAGGTACGGTACCGTTGTTGGCGCGTCGCGTGC3'
```

NARF-Seed2

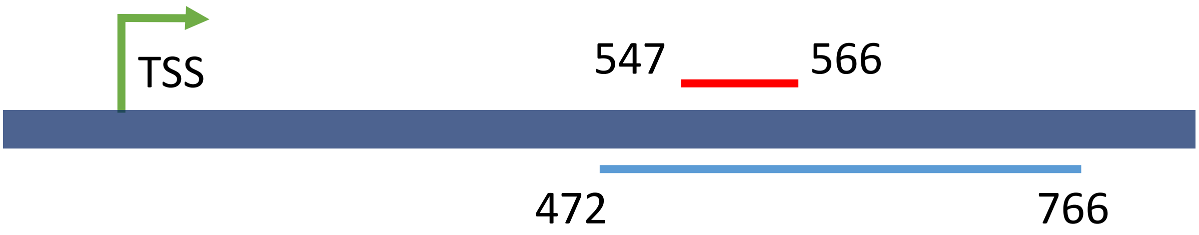

Region	Genome coordinates (hg19)
SNP	chr17:80417150-80417150
sgRNA	chr17:80417135-80417154 (+)
NGS analysis region	chr17:80417054-80417348

NARF-Seed2 sgRNA designed to target Allele 2

sgRNA

CCGCGGTTGTCTCCATGCCA

Allele 1

```
5'CGGCTGCTGACCGGCGCCTTCCGCGGTTGTCTCCAGGCCATGGCAACAACCGCGCAGCGCACG5'
  |||||||||||||||||||||||||||||||||||||||||||||||||||||||||||||||
3'GCCGACGACTGGCCGCGGAAGGCGCCAACAGAGGTCCGGTACCGTTGTTGGCGCGTCGCGTGC3'
```

Allele 2

```
                  CCGCGGTTGTCTCCATGCCA
5'CGGCTGCTGACCGGCGCCTTCCGCGGTTGTCTCCATGCCATGGCAACAACCGCGCAGCGCACG5'
  |||||||||||||||||||||||||||||||||||||||||||||||||||||||||||||||
3'GCCGACGACTGGCCGCGGAAGGCGCCAACAGAGGTACGGTACCGTTGTTGGCGCGTCGCGTGC3'
```

PDE8A-PAM2

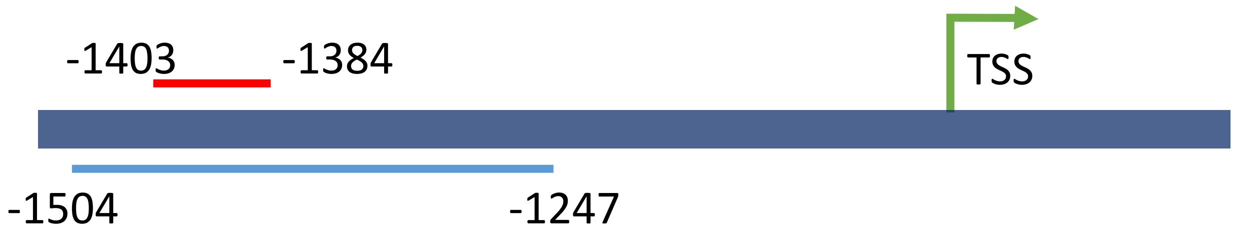

Region	Genome coordinates (hg19)
SNP	chr15:85523678-85523678
sgRNA	chr15:85523657-85523676 (+)
NGS analysis region	chr15:85523556-85523813

PDE8A-PAM2 sgRNA designed to target Allele 1

sgRNA

CTCCGGGTCTTTGCAGTAGC

Allele 1

```
                    CTCCGGGTCTTTGCAGTAGC
5'GAATCCAGCCCGCAGCACGCCTCCGGGTCTTTGCAGTAGCCGGGCCAAGGCGGGAGCGGCTGC3'
  |||||||||||||||||||||||||||||||||||||||||||||||||||||||||||||||
3'CTTAGGTCGGGCGTCGTGCGGAGGCCCAGAAACGTCATCGGCCCGGTTCCGCCCTCGCCGACG5'
```

Allele 2

```
5'GAATCCAGCCCGCAGCACGCCTCCGGGTCTTTGCAGTAGCCTGGCCAAGGCGGGAGCGGCTGC3'
  |||||||||||||||||||||||||||||||||||||||||||||||||||||||||||||||
3'CTTAGGTCGGGCGTCGTGCGGAGGCCCAGAAACGTCATCGGACCGGTTCCGCCCTCGCCGACG5'
```

PDE8A-Seed1

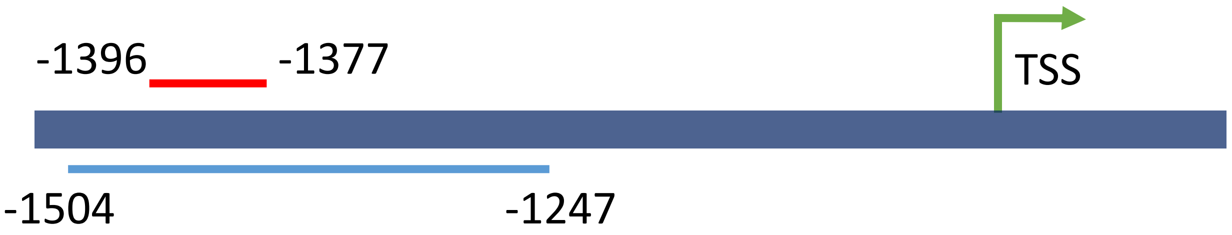

Region	Genome coordinates (hg19)
SNP	chr15:85523678-85523678
sgRNA	chr15:85523664-85523683 (+)
NGS analysis region	chr15:85523556-85523813

PDE8A-Seed1 sgRNA designed to target Allele 1

sgRNA

TCTTTGCAGTAGCCGGGCCA

Allele 1

```
                     TCTTTGCAGTAGCCGGGCCA
5'GCCCGCAGCACGCCTCCGGGTCTTTGCAGTAGCCGGGCCAAGGCGGGAGCGGCTGCCTCTCAG3'
  |||||||||||||||||||||||||||||||||||||||||||||||||||||||||||||||
3'CGGGCGTCGTGCGGAGGCCCAGAAACGTCATCGGCCCGGTTCCGCCCTCGCCGACGGAGAGTC5'
```

Allele 2

```
5'GCCCGCAGCACGCCTCCGGGTCTTTGCAGTAGCCTGGCCAAGGCGGGAGCGGCTGCCTCTCAG3'
  |||||||||||||||||||||||||||||||||||||||||||||||||||||||||||||||
3'CGGGCGTCGTGCGGAGGCCCAGAAACGTCATCGGACCGGTTCCGCCCTCGCCGACGGAGAGTC5'
```

PDE8A-Seed2

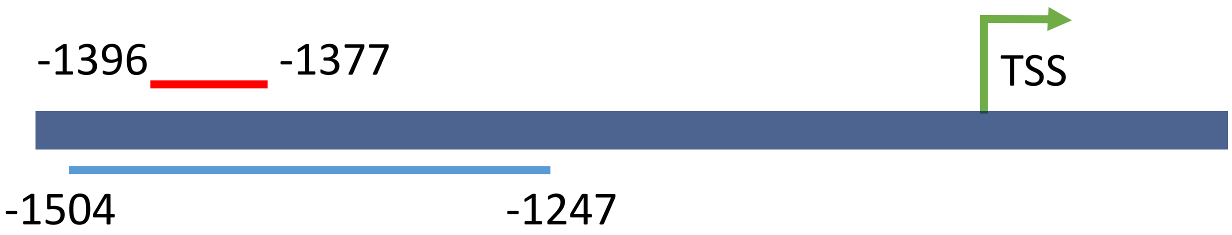

Region	Genome coordinates (hg19)
**SNP**	chr15:85523678-85523678
**sgRNA**	chr15:85523664-85523683 (+)
**NGS analysis region**	chr15:85523556-85523813

PDE8A-Seed2 sgRNA designed to target Allele 2

sgRNA

TCTTTGCAGTAGCCGGGCCA

Allele 1

```
5'GCCCGCAGCACGCCTCCGGGTCTTTGCAGTAGCCGGGCCAAGGCGGGAGCGGCTGCCTCTCAG3'
  |||||||||||||||||||||||||||||||||||||||||||||||||||||||||||||||
3'CGGGCGTCGTGCGGAGGCCCAGAAACGTCATCGGCCCGGTTCCGCCCTCGCCGACGGAGAGTC5'
```

Allele 2

```
                    TCTTTGCAGTAGCCTGGCCA
5'GCCCGCAGCACGCCTCCGGGTCTTTGCAGTAGCCTGGCCAAGGCGGGAGCGGCTGCCTCTCAG3'
  |||||||||||||||||||||||||||||||||||||||||||||||||||||||||||||||
3'GTGAGGCAAGGTATGCCAAGGGTAGAGTTCACCCATTGACGCCTGAGGTCTCGTCCCGTCCCC5'
```

RAF1-PAM3

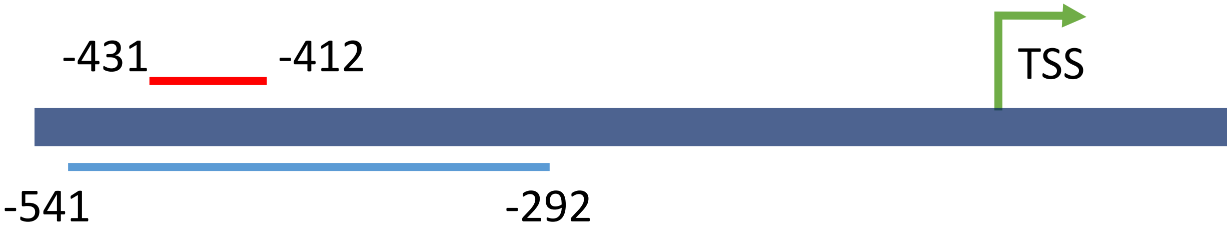

Region	Genome coordinates (hg19)
**SNP**	chr3:12705208-12705208
**sgRNA**	chr3:12705186-12705205 (+)
**NGS analysis region**	chr3:12705076-12705325

RAF1-PAM3 sgRNA designed to target Allele 1

sgRNA

AGCCCTCTGCCCGGCAGCCG

Allele 1

```
                    AGCCCTCTGCCCGGCAGCCG
5‘GGTGACAACGGCCTGGCCCAAGCCCTCTGCCCGGCAGCCGCGGGGCCGCTCCATCAGCGCCAC3‘
3‘CCACTGTTGCCGGACCGGGTTCGGGAGACGGGCCGTCGGCGCCCCGGCGAGGTAGTCGCGGTG5‘
```

Allele 2

```
5‘GGTGACAACGGCCTGGCCCAAGCCCTCTGCCCGGCAGCCGCGAGGCCGCTCCATCAGCGCCAC3‘
3‘CCACTGTTGCCGGACCGGGTTCGGGAGACGGGCCGTCGGCGCTCCGGCGAGGTAGTCGCGGTG5‘
```

RALB-PAM2

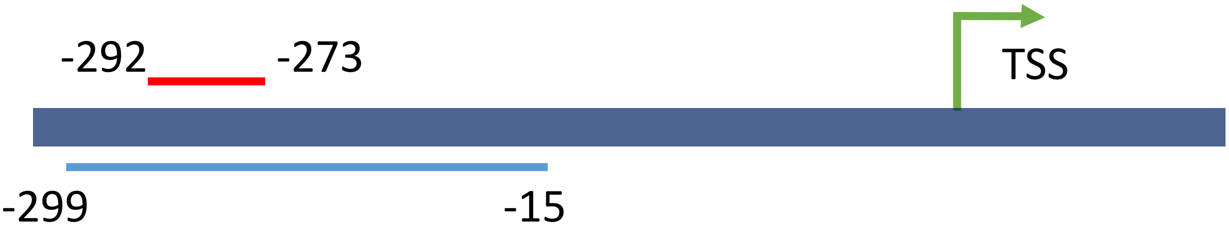

Region	Genome coordinates (hg19)
SNP	chr2:121010211-121010211
sgRNA	chr2:121010190-121010209 (+)
NGS analysis region	chr2:121010183-121010467

RALB-PAM2 sgRNA designed targeting Allele 1

sgRNA

GAGAGAGAAACAGAGGGGGA

Allele 1

```
                   GAGAGAGAAACAGAGGGGGA
5‘AGCTGCGTGTCCGAGGGGCAGAGAGAGAAACAGAGGGGGACGGGGCAGGCGCGCTGGGCCCGC3‘
  |||||||||||||||||||||||||||||||||||||||||||||||||||||||||||||||
3‘TCGACGCACAGGCTCCCCGTCTCTCTCTTTGTCTCCCCCTGCCCCGTCCGCGCGACCCGGGCG5‘
```

Allele 2

```
5‘AGCTGCGTGTCCGAGGGGCAGAGAGAGAAACAGAGGGGGACCGGGCAGGCGCGCTGGGCCCGC3‘
  |||||||||||||||||||||||||||||||||||||||||||||||||||||||||||||||
3‘ACTTCCACTTGACGACTGATTTCAAACCAGGGAAAGCGAGGGAGAGGAACGCTTTTACGAGAT3‘
```

TTC41P-PAM3

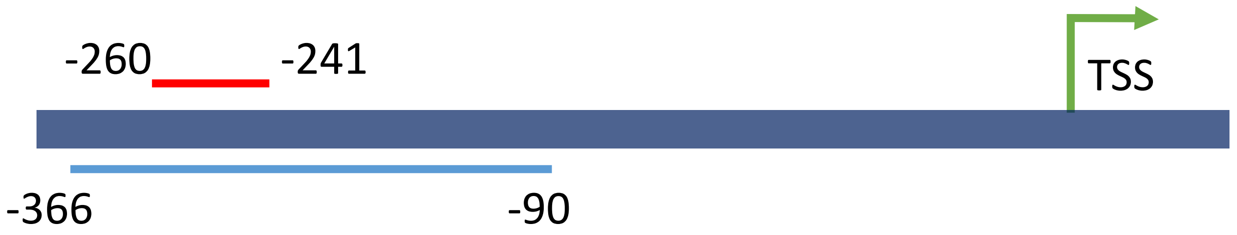

Region	Genome coordinates (hg19)
**SNP**	chr12:104323950-104323950
**sgRNA**	chr12:104323928-104323947 (+)
**NGS analysis region**	chr12:104323822-104324098

TTC41P-PAM3 sgRNA designed to target Allele 1

sgRNA

AAGGGGCGGTCCCACGTGTG

Allele 1

```
                    AAGGGGCGGTCCCACGTGTG
5'AGGCCAAGGCCGCCCAGGCAAAGGGGCGGTCCCACGTGTGAGGGGCCCGCGGAGCCATTTGAT3'
  |||||||||||||||||||||||||||||||||||||||||||||||||||||||||||||||
3'TCCGGTTCCGGCGGGTCCGTTTCCCCGCCAGGGTGCACACTCCCCGGGCGCCTCGGTAAACTA5'
```

Allele 2

```
5'AGGCCAAGGCCGCCCAGGCAAAGGGGCGGTCCCACGTGTGAGTGGCCCGCGGAGCCATTTGAT3'
  |||||||||||||||||||||||||||||||||||||||||||||||||||||||||||||||
3'TCCGGTTCCGGCGGGTCCGTTTCCCCGCCAGGGTGCACACTCACCGGGCGCCTCGGTAAACTA5'
```

TYK2-PAM2

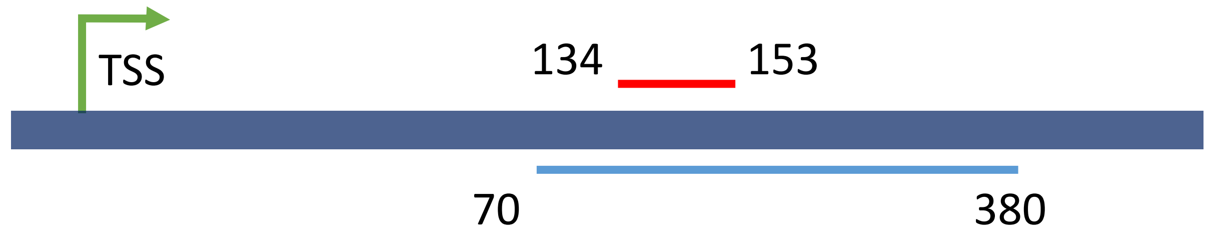

Region	Genome coordinates (hg19)
**SNP**	chr19:10491382-10491382
**sgRNA**	chr19:10491361-10491380 (+)
**NGS analysis region**	chr19:10491297-10491607

TYK2-PAM2 sgRNA designed to target Allele 1

sgRNA

GGCGGCACTCTCATTGGTCC

Allele 1

```
                           GGCGGCACTCTCATTGGTCC
5'TTTTTTTTTTTTGCCATGGTGGCGGCACTCTCATTGGTCCTGGGGCTCATTCGTCACTCAGCC3'
  ||||||||||||||||||||||||||||||||||||||||||||||||||||||||||||||||
3'AAAAAAAAAAAACGGTACCACCGCCGTGAGAGTAACCAGGACCCCGAGTAAGCAGTGAGTCGG5'
```

Allele 2

```
5'TTTTTTTTTTTTGCCATGGTGGCGGCACTCTCATTGGTCCTCGGGCTCATTCGTCACTCAGCC3'
  ||||||||||||||||||||||||||||||||||||||||||||||||||||||||||||||||
3'AAAAAAAAAAAACGGTACCACCGCCGTGAGAGTAACCAGGAGCCCGAGTAAGCAGTGAGTCGG5'
```

sgRNA binding sites within the analyzed target regions and corresponding CpG site numbers

Category	Experiment	CpG site numbers
**Category 1**	DAP3-Seed1	3, 4, 5
	DAP3-Seed2	3, 4, 5
	GSPT1-Seed1	7
	GSPT1-Seed2	7
	ISG15-Seed1	16, 17, 18
	ISG15-Seed2	16, 17, 18
	MAPK1-Seed1	11, 12, 13
	MYH10-Seed1	6
	NARF-Seed1	8, 9
	NARF-Seed2	8, 9
	PDE8A-Seed1	9, 10
	PDE8A-Seed2	9, 10
**Category 2**	ISG15-PAM2	16, 17, 18
	NARF-PAM2	7, 8, 9
	PDE8A-PAM2	8, 9
	RALB-PAM2	1
	TYK2-PAM2	2
**Category 3**	DAP3-PAM3	3, 4, 5
	GPD1L-PAM3	5, 6
	GSPT1-PAM3	6
	MRPL52-PAM3	between 5 and 6
	MSH6-PAM3	in front of 1
	RAF1-PAM3	9, 10, 11
	TTC41P-PAM3	7, 8
