# Supplementary material for: Development of super-specific epigenome editing by targeted allele-specific DNA methylation
Source: Epigenetics Chromatin. 2023 Oct 21;16:41. doi: 10.1186/s13072-023-00515-5 (PMC10589950; doi:10.1186/s13072-023-00515-5)

Development of super-specific epigenome editing by targeted allele-specific DNA methylation

Additional file 2

Supplementary figures

Supplementary Figure 1. Overview of the workflow for ASM applied here.

Supplementary Figure 2. DNA methylation observed after targeting a SNP in the seed region of the sgRNA for additional experiments with weak or no ASM.

Supplementary Figure 3. DNA methylation profiles observed at the VEGFA locus as an off-target locus control.

Supplementary Figure 4. DNA methylation observed after targeting a SNP at the second PAM position for additional experiments with weak or no ASM.

Supplementary Figure 5. DNA methylation profiles observed after targeting a SNP at the third PAM position for experiments with weak or no ASM.

Supplementary Figure 6. Stability of BFP, sfGFP and DsRed observed in the experiments investigating the stability of ASM.

Supplementary Figure 7. DNA methylation profiles observed in the experiments investigating the stability of ASM.

Supplementary Figure 8: Gating strategy used for the sorting of cells containing all three plasmids which encode the components of the EpiEditing complex and co-express BFP, sfGFP and DsRed.

Supplementary Figure 1. Overview of the workflow for ASM applied here. The dCas9-10x SunTag, scFv-DNMT3A/3L, multi-sgRNA plasmids were transfected into HEK293 cells. They co-express BFP, sfGFP and DsRed, respectively, which was used on the third day of transfection to sort the triple-positive cells by FACS. Genomic DNA was isolated from the sorted cells, subjected to bisulfite treatment and used for library generation and NGS followed by data analysis.

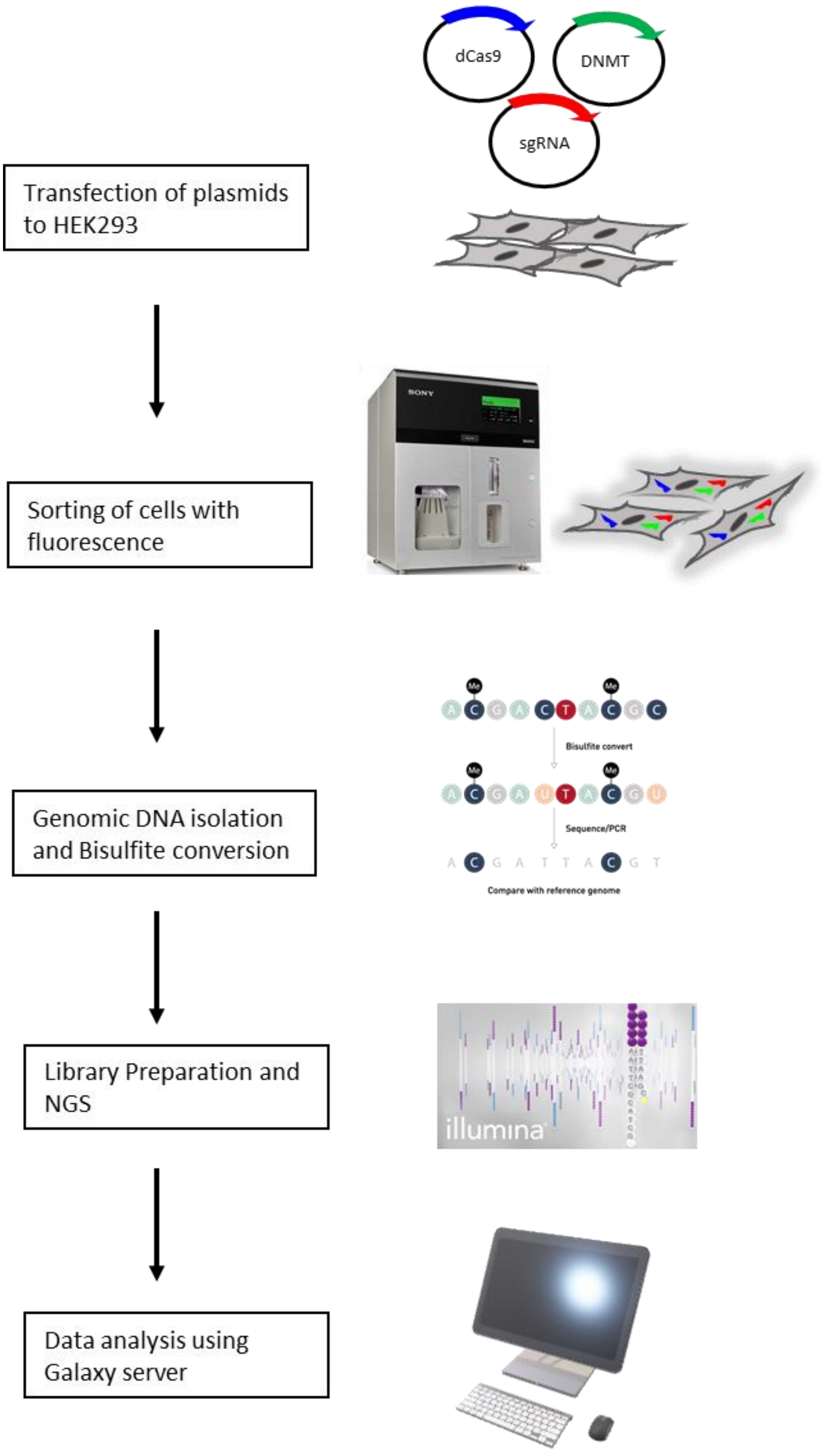

Supplementary Figure 2. DNA methylation observed after targeting a SNP in the seed region of the sgRNA for additional experiments with weak or no ASM. The graphs show the DNA methylation at each CpG site in the different target regions. The CpG sites in the grey regions were included for the calculation of the respective summary bar graphs. “scr-sg” refers to samples treated with scrambled sgRNA, “gene-sg” to samples treated with the allele-specific sgRNA. The sgRNA binding site is indicated by a dark red line. The bar graphs show corresponding levels of average DNA methylation at the selected CpG sites. Error bars show the standard deviation (SD) of three independent biological repeats except for MAPK1-Seed1 which was conducted twice.

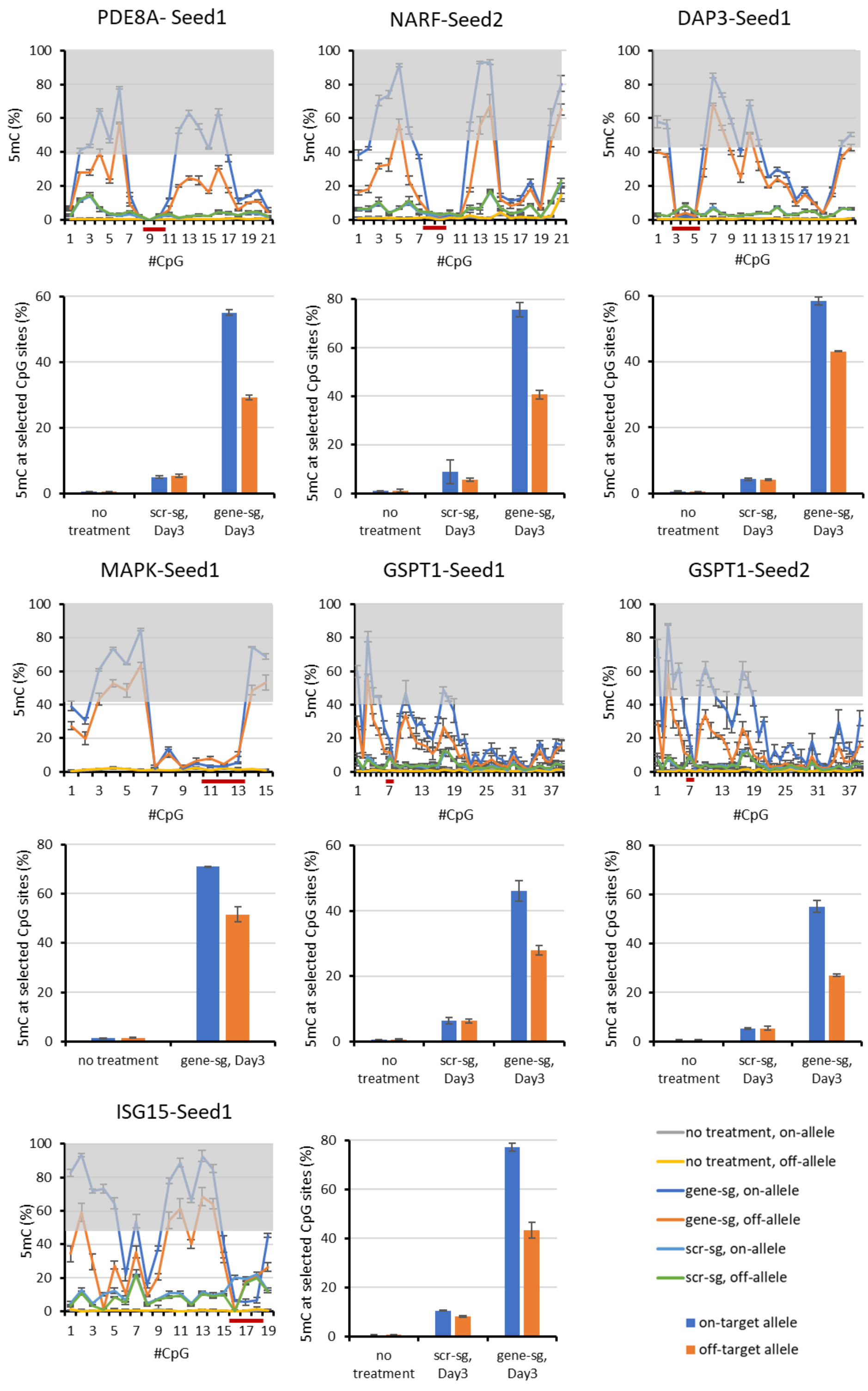

Supplementary Figure 3. DNA methylation profiles observed at the VEGFA locus as an off-target locus control. The bar diagrams show the average DNA methylation at all 12 CpG sites of the VEGFA region (chr6:43737633-43739852, hg19). **A)** VEGFA locus methylation observed in the experiments shown in Figure 2-4. Error bars show the SD of three independent biological repeats except for Multiguide 1 and Multiguide 2 where two repeats were conducted. The composition of the multiguide sgRNAs is indicated. **B)** VEGFA locus methylation observed in the experiments shown in Figure 6. Error bars show the SD of three independent biological repeats except for Multiguide 1, that was repeated twice.

A

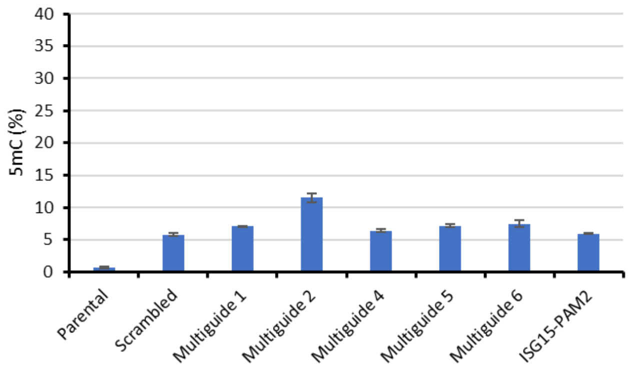

Multiguide 1- ISG15-Seed2, MSH6-PAM3, MAPK1-Seed1, MYH10-Seed1
Multiguide 2- ISG15-Seed2, TYK2-PAM2, RAF1-PAM3, RALB-PAM2
Multiguide 3- GPD1L-PAM3, TTC41P-PAM3, MRPL52-PAM3, PDE8A-PAM2
Multiguide 4- GSPT1-PAM3, NARF-PAM2, DAP3-PAM3
Multiguide 5- GSPT1-Seed1, NARF-Seed1, DAP3-Seed1, PDE8A-Seed1
Multiguide 6- GSPT1-Seed2, NARF-Seed2, DAP3-Seed2, PDE8A-Seed2

B

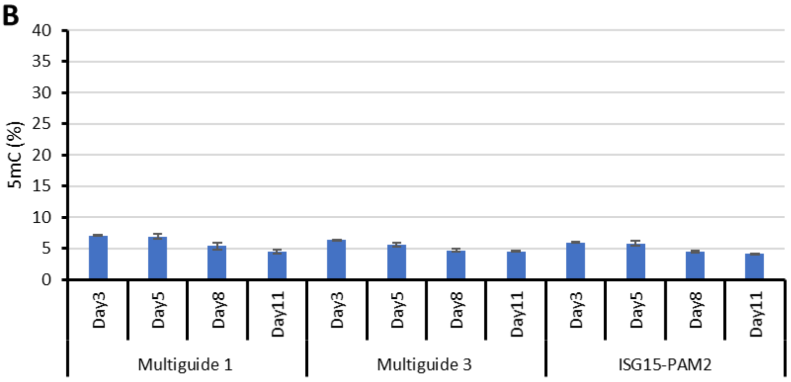

Supplementary Figure 4. DNA methylation observed after targeting a SNP at the second PAM position for additional experiments with weak or no ASM. The graphs show the DNA methylation at each CpG site in the different target regions. The CpG sites in the grey regions were included for the calculation of the respective summary bar graphs. “gene-sg” refers to samples treated with the allele-specific sgRNA. The sgRNA binding site is indicated by a dark red line. The bar graphs show corresponding levels of average DNA methylation at the selected CpG sites. Error bars show the standard deviation (SD) of two independent biological repeats.

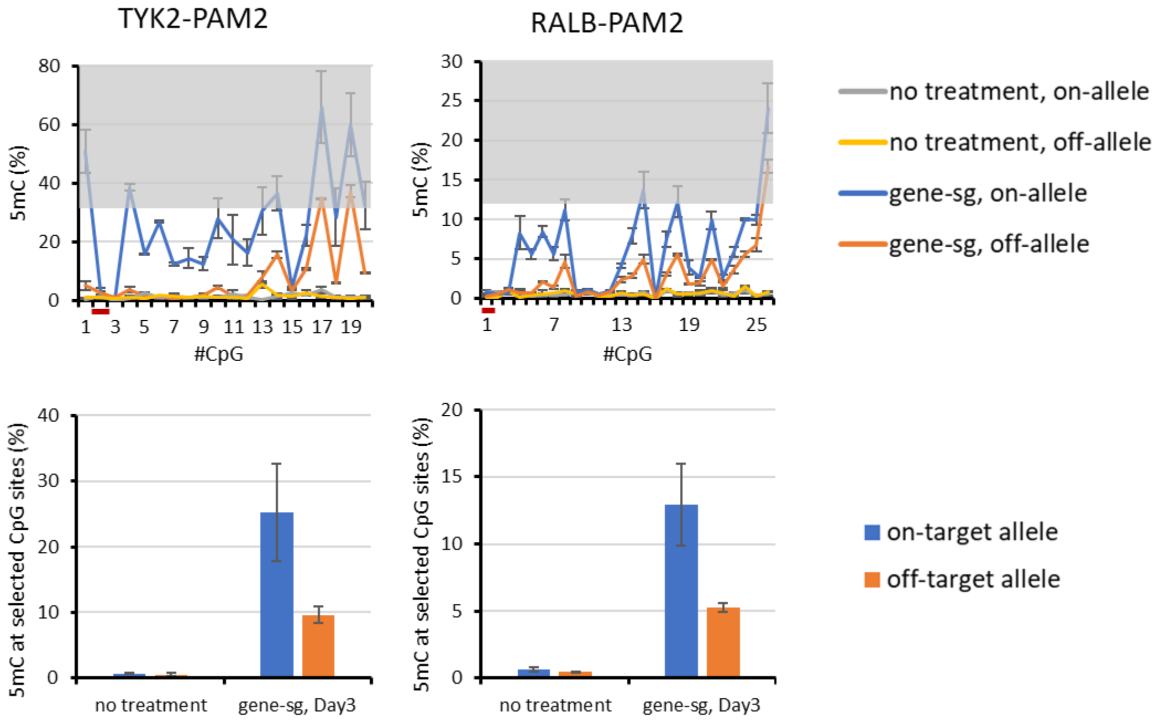

Supplementary Figure 5. DNA methylation profiles observed after targeting a SNP at the third PAM position for experiments with weak or no ASM. The graphs show the DNA methylation at each CpG site in the different target regions. The CpG sites in the grey regions were included for the calculation of the respective summary bar graphs. “gene-sg” refers to samples treated with the allele-specific sgRNA. The sgRNA binding site is indicated by a dark red line. The bar graphs show corresponding levels of average DNA methylation at the selected CpG sites. Error bars show the SD of three independent biological repeats for TTC41P-PAM3 and two for RAF1-PAM3.

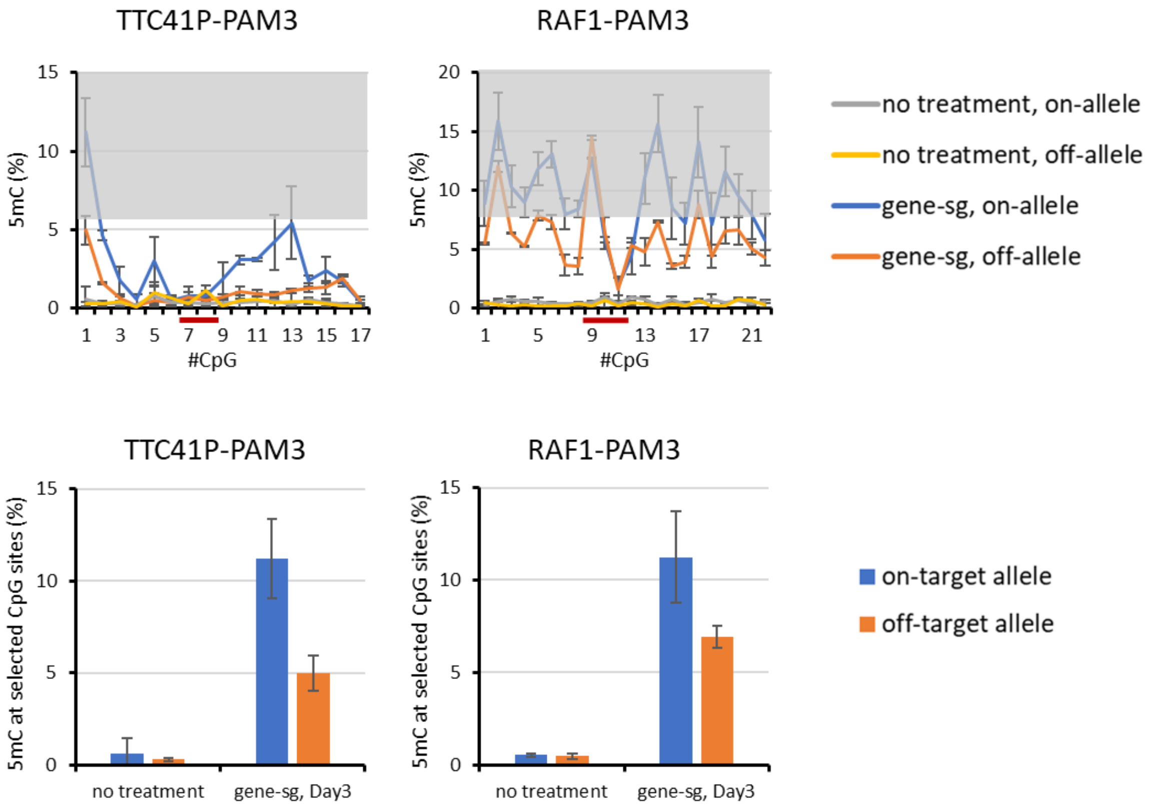

Supplementary Figure 6. Stability of BFP, sfGFP and DsRed observed in the experiments investigating the stability of ASM. The graphs show the fluorescence signals of BPF, sfGFP and DsRed in the sample targeted with a MultiguideRNA1 containing ISG15-Seed2, MSH6-PAM3, MAPK1-Seed1, and MYH10-Seed1. Triple positive cells were sorted on Day 3 and maintained until Day 11 and the corresponding fluorescence levels determined by flow cytometry.

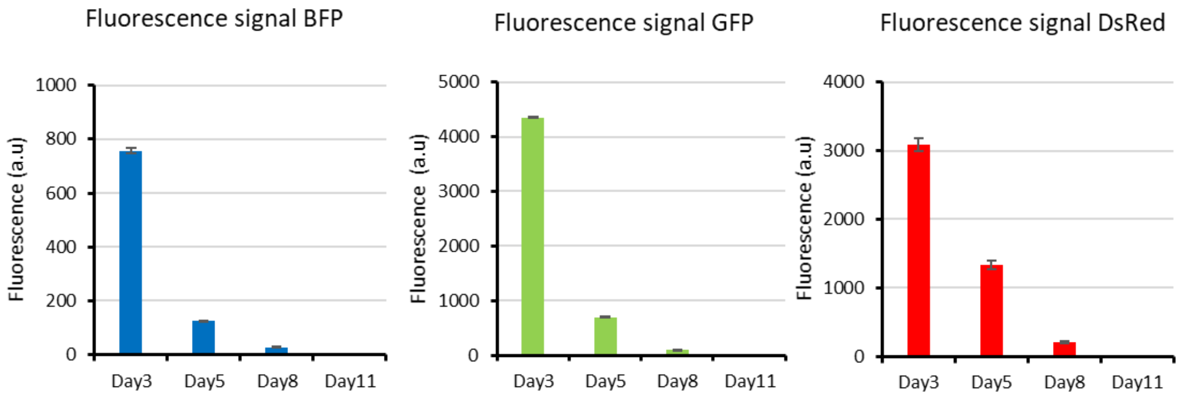

Supplementary Figure 7. DNA methylation profiles observed in the experiments investigating the stability of ASM. The graph indicates the percentage of DNA methylation at each CpG site in the different target regions. The CpG sites in the grey regions were included for the calculation of the respective summary bar graphs. "scr-sg" refers to samples treated with scrambled sgRNA, "gene-sg" to samples treated with the allele-specific sgRNA. Error bars show the SD of three independent biological repeats except ISG15-Seed2, MSH6-PAM3 and MYH10-Seed1 which were conducted twice. Compiled data are shown in Figure 6.

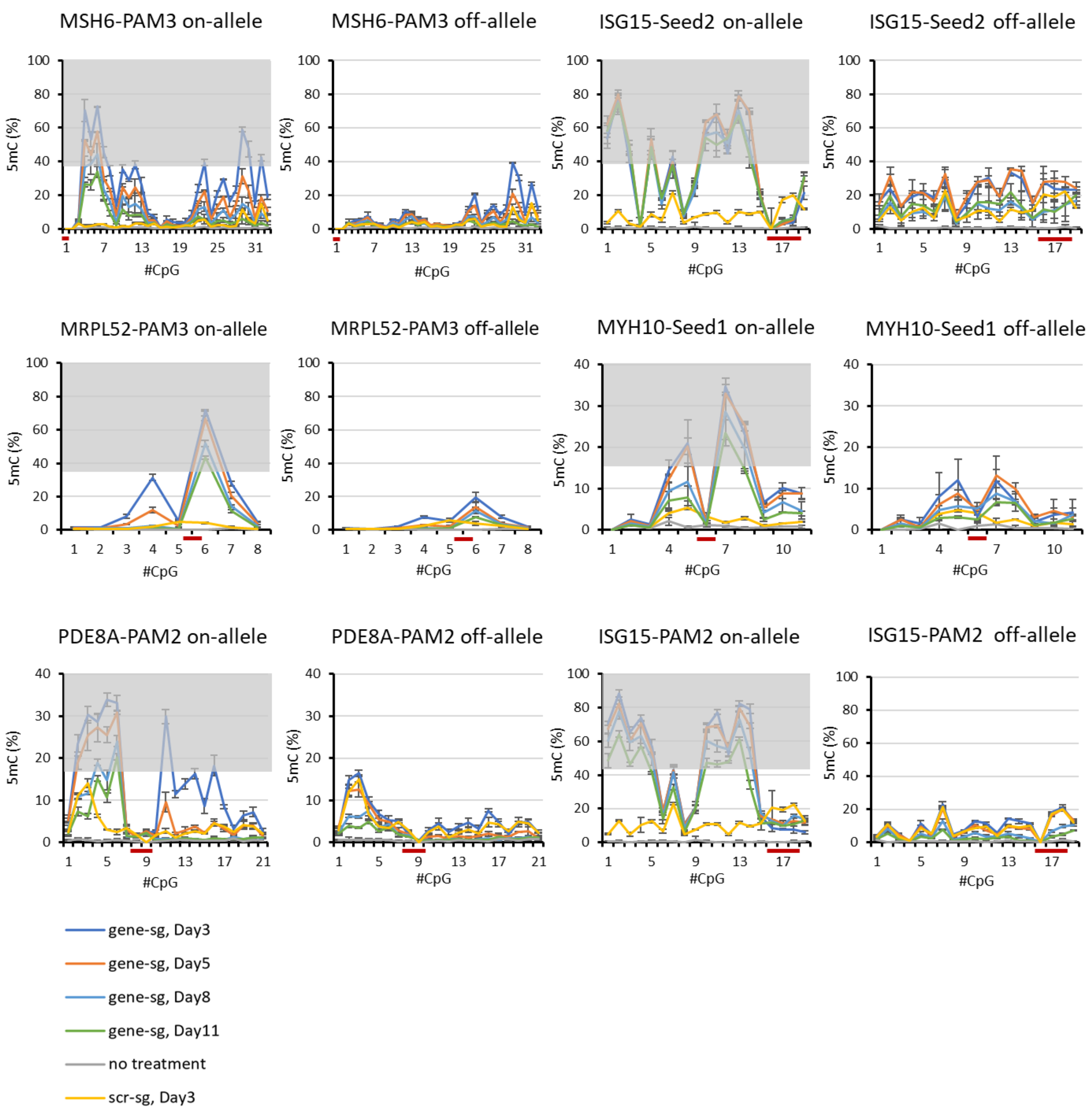

Supplementary Figure 8: Gating strategy used for the sorting of cells containing all three plasmids which encode the components of the EpiEditing complex and co-express BFP, sfGFP and DsRed. FACS sorting was conducted at day 3 after transfection. BSC-A, back scatter area; FSC-A forward scatter area; FSH-H, forward scatter height.

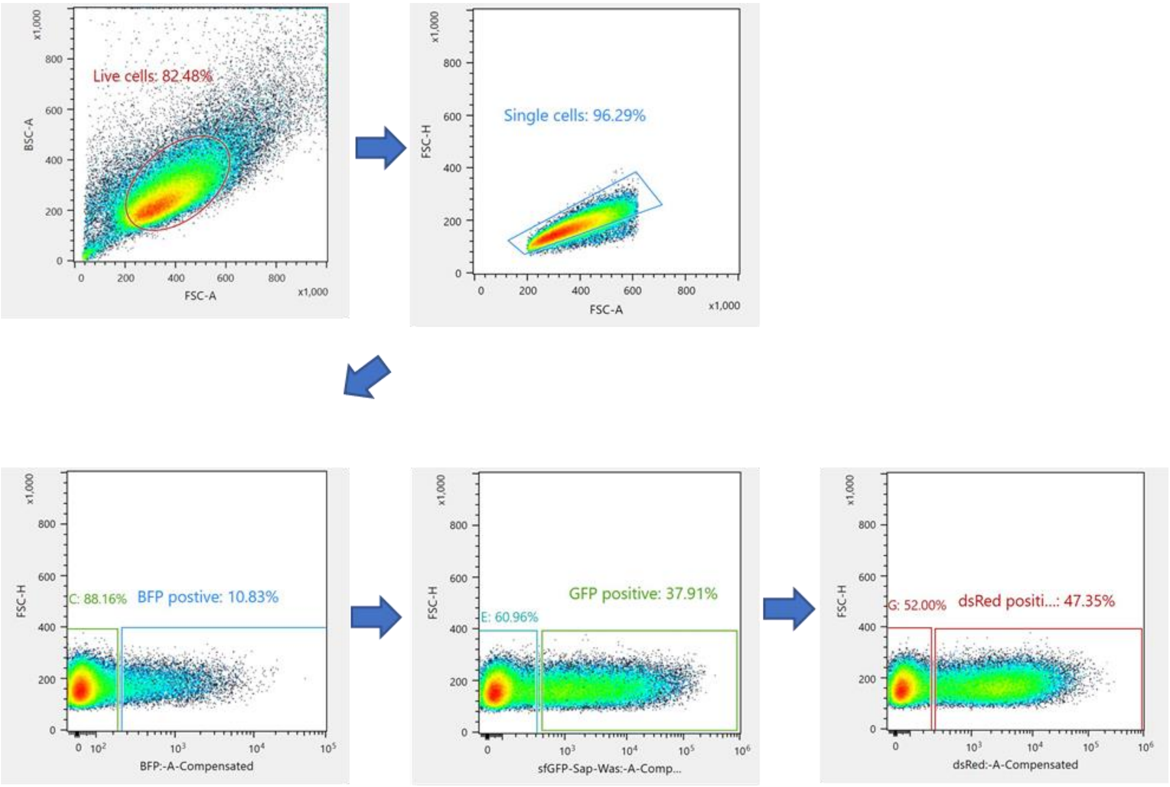

Supplement: Supplementary file 2 — Additional file 2: Fig. S1. Overview of the workflow for ASM applied here. Fig. S2. DNA methylation observed after targeting a SNP in the seed region of the sgRNA for additional experiments with weak or no ASM. Fig. S3. DNA methylation profiles observed at the VEGFA locus as an off-target locus control. Fig. S4. DNA methylation observed after targeting a SNP at the second PAM position for additional experiments with weak or no ASM. Fig. S5. DNA methylation profiles observed after targeting a SNP at the third PAM position for experiments with weak or no ASM. Fig. S6. Stability of BFP, sfGFP and DsRed observed in the experiments investigating the stability of ASM. Fig. S7. DNA methylation profiles observed in the experiments investigating the stability of ASM. Fig. S8. Gating strategy used for the sorting of cells containing all three plasmids which encode the components of the EpiEditing complex and co-express BFP, sfGFP and DsRed. [file 13072_2023_515_MOESM2_ESM.pdf]
