# Supplementary material for: Development of super-specific epigenome editing by targeted allele-specific DNA methylation
Source: Epigenetics Chromatin. 2023 Oct 21;16:41. doi: 10.1186/s13072-023-00515-5 (PMC10589950; doi:10.1186/s13072-023-00515-5)
Supplement: Supplementary file 3 — Additional file 3: Table S1. List of oligodeoxynucleotide used in sgRNA cloning. Table S2. List of primers used for multi-sgRNA cloning. Table S3. List of target specific primers used for amplifying bisulfite treated gDNA samples. Table S4. List of Illumina specific primers used in the second PCR reaction. Table S5. List of primers used for transcript amplification from cDNA. [file 13072_2023_515_MOESM3_ESM.pdf]

Development of super-specific epigenome editing by targeted allele-specific DNA methylation

Additional file 3

Supplementary tables

Supplementary Table 1: List of oligodeoxynucleotide used in sgRNA cloning.

Supplementary Table 2: List of primers used for multi-sgRNA cloning.

Supplementary Table 3: List of target specific primers used for amplifying bisulfite treated gDNA samples.

Supplementary Table 4: List of Illumina specific primers used in the second PCR reaction.

Supplementary Table 5: List of primers used for transcript amplification from cDNA.

Supplementary Table 1: List of oligodeoxynucleotide used in sgRNA cloning.

**Gene**	**sgRNA**	**Description**
DAP3	accgGCAGCGTGTGTCGGTCGCCT	DAP3-PAM3 sgRNA sense oligo
DAP3	aaacAGGCGACCGACACACGCTGC	DAP3-PAM3 sgRNA antisense oligo
DAP3	accgCGTGTGTCGGTCGCCTAGGC	DAP3-Seed1 sgRNA sense oligo
DAP3	aaacGCCTAGGCGACCGACACACG	DAP3-Seed1 sgRNA antisense oligo
DAP3	accgCGTGTGTCGGTCGCCTAGTC	DAP3-Seed2 sgRNA sense oligo
DAP3	aaacGACTAGGCGACCGACACACG	DAP3-Seed2 sgRNA antisense oligo
GPD1L	accgCTTGCTGCCCAGGTCACGGG	GPD1L-PAM3 sgRNA sense oligo
GPD1L	aaacCCCGTGACCTGGGCAGCAAG	GPD1L-PAM3 sgRNA antisense oligo
GSPT1	accgTCCATACGGTTCCCATCTCA	GSPT1-PAM3 sgRNA sense oligo
GSPT1	aaacTGAGATGGGAACCGTATGGA	GSPT1-PAM3 sgRNA antisense oligo
GSPT1	accgCCATCTCAAGGGGGTAACTG	GSPT1-Seed11 sgRNA sense oligo
GSPT1	aaacCAGTTACCCCCTTGAGATGG	GSPT1-Seed1 sgRNA antisense oligo
GSPT1	accgCCATCTCAAGTGGGTAACTG	GSPT1-Seed2 sgRNA sense oligo
GSPT1	aaacCAGTTACCCACTTGAGATGG	GSPT1-Seed2 sgRNA antisense oligo
ISG15	accgTGCCTCTCAGCCGCCGGCTT	ISG15-PAM2 sgRNA sense oligo
ISG15	aaacAAGCCGGCGGCTGAGAGGCA	ISG15-PAM2 sgRNA antisense oligo
ISG15	accgTCTCAGCCGCCGGCTTCGGC	ISG15-Seed1 sgRNA sense oligo
ISG15	aaacGCCGAAGCCGGCGGCTGAGA	ISG15-Seed1 sgRNA antisense oligo
ISG15	accgTCTCAGCCGCCGGCTTCCGC	ISG15-Seed2 sgRNA sense oligo
ISG15	aaacGCGGAAGCCGGCGGCTGAGA	ISG15-Seed2 sgRNA antisense oligo
MAPK1	accgGCTGCGGCGCTGCCGGGATA	MAPK1-Seed1 sgRNA sense oligo
MAPK1	aaacTATCCCGGCAGCGCCGCAGC	MAPK1-Seed1 sgRNA antisense oligo
MRPL52	accgCTGCTCAGCATGGCTGCTTT	MRPL52-PAM3 sgRNA sense oligo
MRPL52	aaacAAAGCAGCCATGCTGAGCAG	MRPL52-PAM3 sgRNA antisense oligo
MSH6	accgAAGTTTGGTCCCTTTCGCTC	MSH6-PAM3 sgRNA sense oligo
MSH6	aaacGAGCGAAAGGGACCAAACTT	MSH6-PAM3 sgRNA antisense oligo
MYH10	accgGAGCTGTAGGGTTTGTGCTG	MYH10-Seed1 sgRNA sense oligo
MYH10	aaacCAGCACAAACCCTACAGCTC	MYH10-Seed1 sgRNA antisense oligo
NARF	accgCGCCTTCCGCGGTTGTCTCC	NARF-PAM2 sgRNA sense oligo
NARF	aaacGGAGACAACCGCGGAAGGCG	NARF-PAM2 sgRNA antisense oligo
NARF	accgCCGCGGTTGTCTCCAGGCCA	NARF-Seed1 sgRNA sense oligo
NARF	aaacTGGCCTGGAGACAACCGCGG	NARF-Seed1 sgRNA antisense oligo
NARF	accgCCGCGGTTGTCTCCATGCCA	NARF-Seed2 sgRNA sense oligo
NARF	aaacTGGCATGGAGACAACCGCGG	NARF-Seed2 sgRNA antisense oligo
PDE8A	accgCTCCGGGTCTTTGCAGTAGC	PDE8A-PAM2 sgRNA sense oligo
PDE8A	aaacGCTACTGCAAAGACCCGGAG	PDE8A-PAM2 sgRNA antisense oligo
PDE8A	accgTCTTTGCAGTAGCCGGGCCA	PDE8A-Seed1 sgRNA sense oligo
PDE8A	aaacTGGCCCGGCTACTGCAAAGA	PDE8A-Seed1 sgRNA antisense oligo
PDE8A	accgTCTTTGCAGTAGCCTGGCCA	PDE8A-Seed2 sgRNA sense oligo
PDE8A	aaacTGGCCAGGCTACTGCAAAGA	PDE8A-Seed2 sgRNA antisense oligo
RAF1	accgAGCCCTCTGCCCGGCAGCCG	RAF1-PAM3sgRNA sense oligo
RAF1	aaacCGGCTGCCGGGCAGAGGGCT	RAF1-PAM3 sgRNA antisense oligo
RALB	accgGAGAGAGAAACAGAGGGGGA	RALB-PAM2 sgRNA sense oligo

RALB	aaacTCCCCCTCTGTTTCTCTCTC	RALB-PAM2 sgRNA antisense oligo
TTC41P	accgAAGGGGCGGTCCCACGTGTG	TTC41P-PAM3 sgRNA sense oligo
TTC41P	aaacCACACGTGGGACCGCCCCTT	TTC41P-PAM3 sgRNA antisense oligo
TYK2	accgGGCGGCACTCTCATTGGTCC	TYK2-Seed1 sgRNA antisense oligo
TYK2	aaacGGACCAATGAGAGTGCCGCC	TYK2-Seed1 sgRNA antisense oligo

Supplementary Table 2: List of primers used for multi-sgRNA cloning.

Primer	Sequence
PB905	GGCTACGAAGACTATGCCCCAAACTCATCAATGTATCT
PB906	TTCTACGAAGACCCCATAAATTTACGAGCTTTCTGG
PB907	GGCTACGAAGACTATATGCCAAACTCATCAATGTATCT
PB908	TTCTACGAAGACCCAGTTAATTTACGAGCTTTCTGG
PB909	GGCTACGAAGACTAAACTCCAAACTCATCAATGTATCT
PB910	TTCTACGAAGACCCGAATAATTTACGAGCTTTCTGG
PB911	GGCTACGAAGACTAATTCCCAAACTCATCAATGTATCT
PB912	TTCTACGAAGACCCCCTAAATTTACGAGCTTTCTGG
PB913	GGCTACGAAGACTATAGGCCAAACTCATCAATGTATCT
PB914	TTCTACGAAGACCCCGTTAATTTACGAGCTTTCTGG
PB915	GGCTACGAAGACTAAACGCCAAACTCATCAATGTATCT
PB916	TTCTACGAAGACCCTCTGAATTTACGAGCTTTCTGG

Supplementary Table 3: List of target specific primers used for amplifying bisulfite treated gDNA samples.

Gene	Primer name	Sequence
DAP3 FP	NR405	ACACTCTTTCCCTACACGACGCTCTTCCGATCTNNNNNATAGATTTTTTTTGTAGTTTTAGGAYG
DAP3 RP	NR406	GTGACTGGAGTTCAGACGTGTGCTCTTCCGATCTGAGAATTAACAACAACAAAAAACAC
GPD1L FP	NR193	ACACTCTTTCCCTACACGACGCTCTTCCGATCTNNNNNCATGGTTAAGGATTTGGTGGAG
GPD1L RP	NR194	GTGACTGGAGTTCAGACGTGTGCTCTTCCGATCTGATCCTATTAAAACTTAAATTTTCTTCCT
GPD1L FP	NR361	ACACTCTTTCCCTACACGACGCTCTTCCGATCTNNNNNATGGGTTAAGGATTTGGTGGAG
GPD1L RP	NR362	GTGACTGGAGTTCAGACGTGTGCTCTTCCGATCTTATCCTATTAAAACTTAAATTTTCTTCCT
GSPT1 FP	NR395	ACACTCTTTCCCTACACGACGCTCTTCCGATCTNNNNNCATTAAAYGTTTATTTAAATGATTTTTG
GSPT1 RP	NR407	GTGACTGGAGTTCAGACGTGTGCTCTTCCGATCTGATAACCCACATAACRTAAAAATACRATC
ISG15 FP	PB372	ACACTCTTTCCCTACACGACGCTCTTCCGATCTNNNNNAGAGCTTAGGTGTTTTTAGGGTGTTGG
ISG15 RP	PB373	GTGACTGGAGTTCAGACGTGTGCTCTTCCGATCTNNNNNAGCATCACAAACTCCTATACTAACAAAAATAAAT
ISG15 FP	PB376	ACACTCTTTCCCTACACGACGCTCTTCCGATCTNNNNNGACATTTAGGTGTTTTTAGGGTGTTGG
ISG15 RP	PB377	GTGACTGGAGTTCAGACGTGTGCTCTTCCGATCTNNNNNGTCGTCACAAACTCCTATACTAACAAAAATAAAT
ISG15 FP	PB495	ACACTCTTTCCCTACACGACGCTCTTCCGATCTNNNNNGTGTATTAGGTGTTTTTAGGGTGTTGG
ISG15 RP	PB496	GTGACTGGAGTTCAGACGTGTGCTCTTCCGATCTNNNNNGTCACCACAAACTCCTATACTAACAAAAATAAAT
MAPK1 FP	NR031	ACACTCTTTCCCTACACGACGCTCTTCCGATCTNNNNNAAGAAGTGTGTATGYGGATTGGTT
MAPK1 RP	NR032	GTGACTGGAGTTCAGACGTGTGCTCTTCCGATCTGAATTTCTACCCCAATACAAAAACCTCTAAAT
MRPL52 FP	NR197	ACACTCTTTCCCTACACGACGCTCTTCCGATCTNNNNNCATTGATTGAAAATTTAGATTTAAGATGG
MRPL52 RP	NR198	GTGACTGGAGTTCAGACGTGTGCTCTTCCGATCTGATACCRAATCAAAAATCCTATACC
MRPL52 FP	NR365	ACACTCTTTCCCTACACGACGCTCTTCCGATCTNNNNNATGGGTTATTTTGTYTGGGTTAGAG
MRPL52 RP	NR366	GTGACTGGAGTTCAGACGTGTGCTCTTCCGATCTTATCRCCCTCCCTAAACTCC
MSH6 FP	PB374	ACACTCTTTCCCTACACGACGCTCTTCCGATCTNNNNNTGTCGGTGAAGGTGAATTGTTGATTAAAG
MSH6 RP	PB375	GTGACTGGAGTTCAGACGTGTGCTCTTCCGATCTNNNNNTCAGCCTACTAAACTCCCCTTCCCTCA

MSH6 FP	PB483	CACTCTTTCCCTACACGACGCTCTTCCGATCTNNNNNC AGATGTGAAGGTGAATTGTTGATTAAAG
MSH6 RP	PB484	GTGACTGGAGTTCAGACGTGTGCTCTTCCGATCTNNN NNCGTGTCTACTAAACTCCCCTTCCCTCA
MYH10 FP	PB687	ACACTCTTTCCCTACACGACGCTCTTCCGATCTNNNNN ACTGCTATTTTTTAGTTTTTTAGGGGTTGTT
MYH10 RP	PB688	GTGACTGGAGTTCAGACGTGTGCTCTTCCGATCTNNN NNGCAGAATAACTCCTAAACTAAAAAATCCC
MYH10 FP	PB707	ACACTCTTTCCCTACACGACGCTCTTCCGATCTNNNNN TGCGTTATTTTTTAGTTTTTTAGGGGTTGTT
MYH10 RP	PB708	GTGACTGGAGTTCAGACGTGTGCTCTTCCGATCTNNN NNACTCTATAACTCCTAAACTAAAAAATCCC
MYH10 FP	PB754	ACACTCTTTCCCTACACGACGCTCTTCCGATCTNNNNN CGCAGTATTTTTTAGTTTTTTAGGGGTTGTT
MYH10 RP	PB755	GTGACTGGAGTTCAGACGTGTGCTCTTCCGATCTNNN NNGACGAATAACTCCTAAACTAAAAAATCCC
NARF FP	NR399	ACACTCTTTCCCTACACGACGCTCTTCCGATCTNNNNN CATGTTTTYGAATTTGTTTTTTTGAGGG
NARF RP	NR400	GTGACTGGAGTTCAGACGTGTGCTCTTCCGATCTGATR AATTCCCCTAAACACAAAT
PDE8A FP	NR199	ACACTCTTTCCCTACACGACGCTCTTCCGATCTNNNNN CATGGTTATTTTGTYTGGGTTAGAG
PDE8A RP	NR200	GTGACTGGAGTTCAGACGTGTGCTCTTCCGATCTGATC RCCCTCCCTAAACTCC
PDE8A FP	NR363	ACACTCTTTCCCTACACGACGCTCTTCCGATCTNNNNN ATGTGATTGAAAATTTAGATTTAAGATGG
PDE8A RP	NR364	GTGACTGGAGTTCAGACGTGTGCTCTTCCGATCTTATA CCRAATCAAAAATCCTATACC
RAF1 FP	NR033	ACACTCTTTCCCTACACGACGCTCTTCCGATCTNNNNN TTAAATATAAGGAAATTTGAGTGGGA
RAF1 RP	NR034	GTGACTGGAGTTCAGACGTGTGCTCTTCCGATCTTATC CTAACTCCCTCAAATAAATAACAAAAC
RALB FP	NR035	ACACTCTTTCCCTACACGACGCTCTTCCGATCTNNNNN AGGGGTAGAGAGAGAAATAGAGGGG
RALB RP	NR036	GTGACTGGAGTTCAGACGTGTGCTCTTCCGATCTATGC CCTTACCAAAAACCAATCC
TTC41P FP	NR195	ACACTCTTTCCCTACACGACGCTCTTCCGATCTNNNNN CATTTTAGGATTYGGAAGTAGTTT
TTC41P RP	NR196	GTGACTGGAGTTCAGACGTGTGCTCTTCCGATCTGATA CCCACRACACCCAATAAA
TYK2 FP	PB644	ACACTCTTTCCCTACACGACGCTCTTCCGATCTNNNNN GCTACCCCATTAACAACTACTCTATTCTCC
TYK2 RP	PB645	GTGACTGGAGTTCAGACGTGTGCTCTTCCGATCTNNN NNTATACGATATGTAGTTGTGTTTTAGGGT
VEGFA FP	PB504	GTGACTGGAGTTCAGACGTGTGCTCTTCCGATCTNNN NNAGAGCGTTTGTTATTTTTTATTTGAAT
VEGFA RP	PB505	ACACTCTTTCCCTACACGACGCTCTTCCGATCTNNNNN AGCATAATCACTCACTTTACCCCTATC
VEGFA FP	PB506	GTGACTGGAGTTCAGACGTGTGCTCTTCCGATCTNNN NNTGTCGGTTTGTTATTTTTTATTTGAAT

VEGFA RP	PB507	ACACTCTTTCCCTACACGACGCTCTTCCGATCTNNNNNTCAGCAATCACTCACTTTACCCCTATC
VEGFA FP	PB518	GTGACTGGAGTTCAGACGTGTGCTCTTCCGATCTNNNNNGACATGTTTGTTATTTTTTATTTGAAT
VEGFA RP	PB519	ACACTCTTTCCCTACACGACGCTCTTCCGATCTNNNNNGTCGTAATCACTCACTTTACCCCTATC
VEGFA FP	PB522	GTGACTGGAGTTCAGACGTGTGCTCTTCCGATCTNNNNNCAGATGTTTGTTATTTTTTATTTGAAT
VEGFA RP	PB523	ACACTCTTTCCCTACACGACGCTCTTCCGATCTNNNNNCGTGTAATCACTCACTTTACCCCTATC

Supplementary Table 4: List of Illumina specific primers used in the second PCR reaction.

Primer name	Sequence
PB346	AATGATACGGCGACCACCGAGATCTACACATTACTCGACACTCTTTC CCTACACGACGCTCTTCCGATCT
PB347	AATGATACGGCGACCACCGAGATCTACACTCCGGAGAACACTCTTTC CCTACACGACGCTCTTCCGATCT
PB348	AATGATACGGCGACCACCGAGATCTACACCGCTCATTACACTCTTTC CCTACACGACGCTCTTCCGATCT
PB349	AATGATACGGCGACCACCGAGATCTACACGAGATTCCACACTCTTTC CCTACACGACGCTCTTCCGATCT
PB350	CAAGCAGAAGACGGCATACGAGATCGAGTAATGTGACTGGAGTTCA GACGTGTGCTCTTCCGATCT
PB351	CAAGCAGAAGACGGCATACGAGATTCTCCGGAGTGACTGGAGTTCA GACGTGTGCTCTTCCGATCT
PB352	CAAGCAGAAGACGGCATACGAGATAATGAGCGGTGACTGGAGTTCA GACGTGTGCTCTTCCGATCT
PB353	CAAGCAGAAGACGGCATACGAGATGGAATCTCGTGACTGGAGTTCA GACGTGTGCTCTTCCGATCT
PB370	AATGATACGGCGACCACCGAGATCTACACATTCAGAAACACTCTTTC CCTACACGACGCTCTTCCGATCT
PB371	CAAGCAGAAGACGGCATACGAGATTTCTGAATGTGACTGGAGTTCAG ACGTGTGCTCTTCCGATCT
PB481	AATGATACGGCGACCACCGAGATCTACACGAATTCGTACACTCTTTC CCTACACGACGCTCTTCCGATCT
PB482	CAAGCAGAAGACGGCATACGAGATACGAATTCGTGACTGGAGTTCA GACGTGTGCTCTTCCGATCT
PB497	AATGATACGGCGACCACCGAGATCTACACCTGAAGCTACACTCTTTC CCTACACGACGCTCTTCCGATCT
PB498	CAAGCAGAAGACGGCATACGAGATAGCTTCAGGTGACTGGAGTTCA GACGTGTGCTCTTCCGATCT
PB516	AATGATACGGCGACCACCGAGATCTACACTAATGCGCACACTCTTTC CCTACACGACGCTCTTCCGATCT
PB517	CAAGCAGAAGACGGCATACGAGATGCGCATTAGTGACTGGAGTTCA GACGTGTGCTCTTCCGATCT
PB520	AATGATACGGCGACCACCGAGATCTACACCGGCTATGACACTCTTTC CCTACACGACGCTCTTCCGATCT
PB521	CAAGCAGAAGACGGCATACGAGATCATAGCCGGTGACTGGAGTTCA GACGTGTGCTCTTCCGATCT
PB611	AATGATACGGCGACCACCGAGATCTACACTCCGCGAAACACTCTTTC CCTACACGACGCTCTTCCGATCT
PB612	CAAGCAGAAGACGGCATACGAGATTTCGCGGAGTGACTGGAGTTCA GACGTGTGCTCTTCCGATCT
PB650	AATGATACGGCGACCACCGAGATCTACACTCTCGCGCACACTCTTTC CCTACACGACGCTCTTCCGATCT
PB651	CAAGCAGAAGACGGCATACGAGATGCGCGAGAGTGACTGGAGTTCA GACGTGTGCTCTTCCGATCT

PB652	AATGATACGGCGACCACCGAGATCTACACAGCGATAGACACTCTTTCCCTACACGACGCTCTTCCGATCT
PB653	CAAGCAGAAGACGGCATACGAGATCTATCGCTGTGACTGGAGTTCAGACGTGTGCTCTTCCGATCT
PB654	AATGATACGGCGACCACCGAGATCTACACTTCCTCCTACACTCTTTCCCTACACGACGCTCTTCCGATCT
PB655	CAAGCAGAAGACGGCATACGAGATAGGAGGAAGTGACTGGAGTTCAGACGTGTGCTCTTCCGATCT
PB659	AATGATACGGCGACCACCGAGATCTACACTGCTTGCTACACTCTTTCCCTACACGACGCTCTTCCGATCT
PB660	CAAGCAGAAGACGGCATACGAGATAGCAAGCAGTGACTGGAGTTCAGACGTGTGCTCTTCCGATCT
PB661	AATGATACGGCGACCACCGAGATCTACACGGTGATGAACACTCTTTCCCTACACGACGCTCTTCCGATCT
PB662	CAAGCAGAAGACGGCATACGAGATTCATCACCGTGACTGGAGTTCAGACGTGTGCTCTTCCGATCT
PB667	AATGATACGGCGACCACCGAGATCTACACAACCTACGACACTCTTTCCCTACACGACGCTCTTCCGATCT
PB668	CAAGCAGAAGACGGCATACGAGATCGTAGGTTGTGACTGGAGTTCAGACGTGTGCTCTTCCGATCT
PB673	AATGATACGGCGACCACCGAGATCTACACGGATCTGAACACTCTTTCCCTACACGACGCTCTTCCGATCT
PB674	CAAGCAGAAGACGGCATACGAGATTCAGATCCGTGACTGGAGTTCAGACGTGTGCTCTTCCGATCT
PB705	AATGATACGGCGACCACCGAGATCTACACTGATCACGACACTCTTTCCCTACACGACGCTCTTCCGATCT
PB706	CAAGCAGAAGACGGCATACGAGATCGTGATCAGTGACTGGAGTTCAGACGTGTGCTCTTCCGATCT
PB732	AATGATACGGCGACCACCGAGATCTACACAAGCGACTACACTCTTTCCCTACACGACGCTCTTCCGATCT
PB733	CAAGCAGAAGACGGCATACGAGATAGTCGCTTGTGACTGGAGTTCAGACGTGTGCTCTTCCGATCT

Supplementary Table 5: List of primers used for transcript amplification from cDNA.

Primer name	Sequence	Gene
NR417	ACACTCTTTCCCTACACGACGCTCTTCCGATCTNN NNNCATAAGAGGCTTGAATTGGCAGT	MSH6 exon FP
NR418	GTGACTGGAGTTCAGACGTGTGCTCTTCCGATCT GATCATCACTGCTTCCTTCCTCC	MSH6 exon RP
NR419	ACACTCTTTCCCTACACGACGCTCTTCCGATCTNN NNNCATCTGCTGGTGGTGGACAAATG	ISG15 exon FP
NR420	GTGACTGGAGTTCAGACGTGTGCTCTTCCGATCT GATCGCAGATTCATGAACACGGT	ISG15 exon FP
NR421	ACACTCTTTCCCTACACGACGCTCTTCCGATCTNN NNNCATGCTTCTACAAGTGACTCGCC	MYH10 exon FP
NR422	GTGACTGGAGTTCAGACGTGTGCTCTTCCGATCT GATGCCCTCATCTCTTCTGCTTC	MYH10 exon FP
NR425	ACACTCTTTCCCTACACGACGCTCTTCCGATCTNN NNNCATCTACCCCGGCTACTCCTG	MRPL52 exon FP
NR426	GTGACTGGAGTTCAGACGTGTGCTCTTCCGATCT GATTGTGACAGCAGTACAACTCG	MRPL52 exon RP
NR427	ACACTCTTTCCCTACACGACGCTCTTCCGATCTNN NNNCATGTGAGAGGCAGTTCATTAGCA	GPD1L exon FP
NR428	GTGACTGGAGTTCAGACGTGTGCTCTTCCGATCT GATGAACACACTCACTCCTCCAC	GPD1L exon FP
NR431	ACACTCTTTCCCTACACGACGCTCTTCCGATCTNN NNNCATTCTGTGCTCAGGGTGATGGA	NARF exon FP
NR432	GTGACTGGAGTTCAGACGTGTGCTCTTCCGATCT GATCTGCCAGCTCTGTTTCACTGTTTG	NARF exon RP
